# Long‐Term Trends in Incidence and Risk Factors for Hemorrhagic Stroke Subtypes Over 24 Years: The South London Stroke Register

**DOI:** 10.1161/JAHA.124.040371

**Published:** 2025-08-12

**Authors:** Xianqi Li, Lu Liu, Ajay Bhalla, Matthew D. L. O'Connell, Charles D. A. Wolfe, Yanzhong Wang

**Affiliations:** ^1^ Department of Population Health Sciences, School of Life Course and Population Sciences King’s College London London United Kingdom; ^2^ NIHR Applied Research Collaboration (ARC) South London London United Kingdom; ^3^ Guy’s and St Thomas NHS Foundation Trust London United Kingdom

**Keywords:** hemorrhagic stroke, incidence trend, intracerebral hemorrhage, SLSR, subarachnoid hemorrhage, Primary Prevention, Risk Factors, Race and Ethnicity, Epidemiology, Intracranial Hemorrhage

## Abstract

**Background:**

Hemorrhagic stroke (HS), including intracerebral and subarachnoid hemorrhage (ICH and SAH), has high mortality and morbidity. We assessed the 24‐year trends in incidence, prestroke risk factors, medications, and demographic patterns in a multiracial and multiethnic population.

**Methods:**

We used 1995 to 2018 data from the South London Stroke Register. The annual incidence rates of HS, ICH, and SAH were standardized to the 2011 England and Wales population and stratified by age, sex, and race or ethnicity. Incidence trends were assessed using Cochran–Armitage tests and Poisson regression. Multiple logistic regressions (with imputed data) assessed shifts in prestroke variables across demographics.

**Results:**

Among 811 patients with ICH and 308 patients with SAH, HS incidence declined by 52% (ICH), 58% (SAH), and 53% overall all demographic groups between 1995 to 2000 and 2013 to 2018 but plateaued or slightly increased from 2007 to 2018. HS incidence per 100 000/year was 19.26 (ICH, 15.35 [95% CI, 13.66–17.19]; SAH, 3.91 [95% CI, 3.07–4.88)] in 2007–2012, and 19.38 (ICH, 15.04 [95% CI, 13.36–16.86]; SAH, 4.34 [95% CI, 3.45–5.35]) in 2013 to 2018. Antiplatelet use declined (OR, 0.10 [95% CI, 0.02–0.44]), and cholesterol‐lowering drugs (OR, 7.8 [95% CI, 3.41–17.81]) and anticoagulants (OR, 5.54 [95% CI, 2.77–11.07]) increased. Diabetes (OR, 1.92 [95% CI, 1.11–3.32]), hypercholesterolemia (OR, 7.8 [95% CI, 3.41–17.81]), and atrial fibrillation (OR, [95% CI, 2.87 1.55–5.3]) increased, and hypertension remained static (OR, 0.89 [95% CI, 0.6–1.32]). Smoking and alcohol use substantially decreased (OR, 0.37 [95% CI, 0.24–0.57]; OR, 0.28 [95% CI, 0.17–0.48], respectively).

**Conclusions:**

HS (ICH and SAH) incidence declined over 50% in 25 years, mainly in the first 12 years. Medication and prestroke comorbidities only partly explain this reduction. Improved prevention and reduced smoking and drinking may have contributed further.

**Nonstandard Abbreviations and Acronyms**
HS: hemorrhagic stroke
ICH: intracerebral hemorrhage
ICH: primary intracerebral hemorrhage
IRR: incidence rate ratio
OAC: oral anticoagulant
OXVASC: Oxford Vascular Study
SLSR: South London Stroke Register

**Research Perspective**

**What Is New?**

- The incidence of hemorrhagic stroke in the South London Stroke Register, standardized by population of England and Wales, declined >50% from 1995 to 2018, over 24 years, with the majority reduction occurring in the first 12 years.
- The decline trend in intracerebral hemorrhage and subarachnoid hemorrhage remains consistent in White and Black populations and those from other racial or ethnic groups.
- The introduction of innovative preventative strategies and improved management of risk factors, such as reduced drinkers and smokers, may contribute to the reduction.

**What Question Should Be Addressed Next?**

- Population‐based study with data on intracerebral hemorrhage location, aneurysmal and nonaneurysmal (including angionegative) subarachnoid hemorrhage can further investigate the trend in subtypes.
- As a register, we include only patients with stroke. Population‐based cohort studies recruiting healthy people with a long follow‐up can better describe the trend of risk factors.

The GBD (Global Burden of Disease) 2019 revealed that ischemic strokes accounted for 62.4% of stroke (approximately 7.63 million), whereas intracerebral hemorrhage (ICH) and subarachnoid hemorrhage (SAH) comprised 27.9% (around 3.41 million) and 9.7% (near 1.18 million), respectively.^1^ It is projected that the absolute burden of ICH will markedly escalate due to growing and aging populations.^2^ This emphasizes that primary prevention continues to be the foremost strategy to reduce the global burden of hemorrhagic stroke (HS) and its associated health consequences.

ICH has the highest mortality of all stroke types and is a leading cause of disability, with >60% of patients dependent after 1 year.^3^ Multiple studies over the past 4 decades have reported a decline in overall stroke incidence in high‐income countries, primarily attributed to a reduction in ischemic stroke occurrence. Nevertheless, studies on ICH incidence have presented a mixed picture. Several studies have indicated a downward trend in ICH incidence,^1^ particularly in individuals <75 years,^4^ and a decrease in hypertension‐related ICH.^5^ Other studies have suggested a stable overall ICH incidence rate^3^, ^6^, ^7^, ^8^ or an increased ICH rate in patients ≥75 years.^5^ Men have also been reported to exhibit higher incidence rates.^6^, ^9^

Ruptured intracranial aneurysm‐induced SAH accounts for a 5% to 10% share in total stroke cases, embodying an extraordinary disease‐specific burden.^10^ Remarkably, half of these strokes occur in people <55 years old, and an initial hemorrhage leads to death in a third of the cases in the initial days to weeks; most survivors endure enduring disabilities or cognitive impairments.^10^ The loss of productive life‐years post SAH at a community level parallels the magnitude observed in ischemic stroke. The worldwide crude SAH incidence was estimated at 7.9 per 100 000 person‐years but demonstrated marked variation with geography, age, and sex.^11^ Recent register‐based or regional studies have presented inconsistent findings regarding SAH incidence reduction over time.^12^, ^13^ If an actual decrease in SAH incidence exists and potential determinants, such as hypertension^14^ and smoking^15^ can be identified at a population‐based level in the long term and across multiethnic populations, it would have substantial implications for devising primary prevention strategies and thus decreasing disease burden in patients with SAH.

Previous studies bear limitations either in demography—predominantly focusing on White populations^4^, ^5^, ^16^, ^17^—or methodology—being dependent on only 2 distinct time points to estimate temporal trends rather than employing continuous epidemiological monitoring.^5^ Moreover, minimal information has been provided about risk profiles in patients with HS.^4^ To address these gaps, we undertook this observational longitudinal population‐based study to explore the trends in incidence and risk factor profile for ICH and SAH over a span of 24 years, leveraging a community‐based register of all cases from a defined multiracial and multiethnic population of south London.

## METHODS

### Date Sharing Statement

Because of the sensitive nature of the data collected for this study, requests to access the data set for academic use should be made to the SLSR (South London Stroke Register) team: https://www.kcl.ac.uk/lsm/research/divisions/hscr/research/groups/stroke/index.aspx.

### Study Cohort

Data were from the SLSR, a population‐based, longitudinal cohort initiated in 1995 to capture all first‐ever stroke cases occurring within a defined inner‐city area of London.^18^ Our research group had conducted a study on long‐term trends in incidence and risk factors for ischemic stroke subtypes between 2000 and 2015.^19^ In this HS study, we used data from 1995 to 2018, categorizing the year of stroke into 4 periods: 1995 to 2000, 2001 to 2006, 2007 to 2012, and 2013 to 2018. The study zone encompasses 22 electoral wards in the northern regions Lambeth and Southwark.^20^ According to the 2011 UK census,^21^ the total population in this area was approximately 357 308, comprising 56% White, 25% Black (14% Black African, 7% Black Caribbean, and 4% other Black), and 18% various other racial or ethnic groups. Detailed insights into the ethnic composition over different time frames can be found in Table S1.

The surveillance methodologies used are detailed elsewhere^18^, ^21^, ^22^ and are briefly recapitulated here. Additional cases from the community were captured through systematic collaboration with general practitioners practicing within the catchment zone.^21^, ^23^ To maximize completeness, a standardized, multisource surveillance protocol was employed, integrating overlapping notification systems. These included emergency department logs, inpatient ward records, neuroimaging databases, mortality and coroner reports, institutional stroke registries, general practitioner electronic records, and direct referrals from hospital and community health care professionals, therapists, and bereavement staff.^20^, ^21^ Previous evaluations of the SLSR using capture–recapture modeling techniques have estimated case ascertainment completeness at approximately 80%, with a range from 75% to 88% across time periods.^18^, ^24^, ^25^

Data collection was conducted prospectively by a trained team comprising research nurses, physicians, and field staff, who ensured high standards of data accuracy and completeness. Whenever possible, patients were evaluated within 48 hours of referral to the SLSR. Information was cross‐verified using both general practitioner records and hospital documentation.^18^ Stroke diagnoses were made following the World Health Organization criteria.^26^ Pathological subtyping was identified using neuroimaging modalities (computed tomography or magnetic resonance imaging), cerebrospinal fluid examination, or autopsy findings,^18^, ^25^ with final diagnostic confirmation by a study clinician. Based on these assessments, patients were assigned to one of the following categories: cerebral infarction, primary ICH (PICH), or SAH; cases lacking sufficient diagnostic evidence were grouped to unclassified. Cases of traumatic SAH and ICH were excluded from this study to focus on nontraumatic causes of HS. Over time, brain imaging coverage among cases of HS improved from 91.79% in 1995 to 2000 to full imaging (100%) in 2013 to 2018, accompanied by a rise in magnetic resonance imaging use from 5.57% to 15.28%.

The initial assessment captured information on

1. Demographics, including age, sex, and self‐identified race or ethnicity, based on the 1991 UK census question.^20^ Racial and ethnic categories were grouped as Black (African, Caribbean, and other), White, and Other (including Asian, Pakistani, Indian, Bangladeshi, Chinese, and unspecified).
2. Prestroke risk factors, such as current versus former/never smoking status, alcohol consumption (≥21 units/week for men, ≥14 units/week for women), physician‐diagnosed hypertension (systolic >140 mm Hg or diastolic >90 mm Hg), diabetes, hypercholesterolemia (defined as total cholesterol ≥6 mmol/L), history of myocardial infarction, transient ischemic attacks, and atrial fibrillation.
3. Medication use before stroke, including prescriptions for antihypertensive agents, glucose‐lowering therapies (oral hypoglycemics or insulin), lipid‐lowering medications, antiplatelet agents, and anticoagulants.

Informed written consent or, when applicable, assent from a legally authorized representative was obtained for all participants unable to consent directly.^18^ The study received ethical approval from the National Health Service Health Research Authority (22/WA/0027) and was previously approved by the ethics committees of Guy's and St Thomas' Hospital, King's College Hospital, Queen's Square, and Westminster Hospital.

### Statistical Analysis

Population denominators were derived from intercensal estimates based on the 1991, 2001, and 2011 UK censuses.^27^, ^28^, ^29^ To approximate the population structure between census years, age‐, sex‐, and race‐ or ethnicity‐specific proportions were included assuming linear trends. During the period from 2004 to 2007, the geographical coverage of the SLSR was expanded, and corresponding population estimates for this extended area were used in rate calculations. Incidence rates were computed by dividing the number of first‐ever, nonduplicate stroke cases by the estimated population in the study area during each defined time window. Annual and 4‐year grouped incidence rates were calculated for the period from January 1, 1995, to December 31, 2018, and expressed per 100 000 population per year. Incidence estimates were reported for over HS, ICH and SAH and then stratified by age (<55 versus ≥55 years), sex, and race or ethnicity. All incidence rates were directly age standardized to the 2011 England and Wales census population. The incidence rate ratio (IRR) was calculated by dividing the incidence rate in 2013 to 2018 by the incidence rate in 1995 to 2000, and their 95% CIs were derived assuming a Poisson distribution. Cochran–Armitage tests and Poisson regression were used to assess the trends.

The year of stroke variable was categorized into 4 levels, each indicating a 6‐year interval from 1995 to 2018, and preliminary trend analyses were conducted by evaluating whether there are significant differences across time periods in the level of each risk factor using chi‐square tests. To assess the changes in prestroke characteristics, multiple logistic regression models were applied with adjustments of age, sex, and race or ethnicity.

Because the magnitude of change in risk factors level may be confounded by age, sex, race, and ethnicity and a correlation between time and race could possibly modify the proportional effect of time, logistic regression models were fitted with simultaneous adjustment for age, sex, race, and ethnicity (handled as appropriate) and a 2‐way interaction terms of race or ethnicity with time (ie, time‐by‐ethnicity) was incorporated. Interaction was regarded as nominally significant if the 2‐sided *P* value was <0.05 after adjustments for multiplicity and backward elimination of nonsignificant terms would be performed.

To reduce potential bias arising from missing data, multiple imputation using chained equations was employed to create 20 imputed data sets. Variables with missing values included hypertension (7.86%), diabetes (7.60%), hypercholesterolemia (25.02%), atrial fibrillation (8.31%), myocardial infarction (8.58%), transient ischemic attack (8.49%), drinking (19.39%), and smoking (18.14%). Missing medication data were also imputed for antihypertensive (11.80%), antidiabetic (7.33%), antiplatelet (22.43%), anticoagulant (15.64%), and lipid‐lowering therapies (19.93%). Each variable was imputed using a binomial model taking age, sex, race, ethnicity, and date of stroke onset as predictors. Final estimates from the imputed data sets were pooled using Rubin's rules,^30^ and the findings remained robust when compared with those from complete‐case models. All statistical procedures were conducted using Stata version 17.

## RESULTS

### Study Population

Between 1995 and 2018, a total of 1119 individuals with HS were identified, corresponding to 7 000 320 person‐years of observation—equivalent to an average annual population of approximately 291 680 within the SLSR catchment area. Among these cases, 811 (72.48%) were classified as ICH and 309 (27.52%) as SAH. The mean age at HS onset was 62.4 years, with women accounting for 46.7% of cases. In terms of racial and ethnic composition, 57.8% were White, 29.8% Black, and 12.4% from other racial or ethnic backgrounds (Table 1). Notable differences between HS subtypes were observed: patients with PICH were older at onset (mean 65.5 years, *P*<0.001) and less likely to be female (43.4%, *P*<0.001).

**Table 1:** Baseline Characteristics of First Ever Hemorrhagic Stroke by Subtypes

	PICH (n=811)	SAH (n=308)	*P* value
Age,y (mean±SD)	65.5±16.2	54.1±16.8	<0.001*
Female sex	352 (43.4)	171 (55.5)	<0.001*
Year of stroke			0.264
1995–2000	251 (30.95)	100 (32.47)	
2001–2006	246 (30.33)	107 (34.74)	
2007–2012	163 (20.10)	49 (15.91)	
2013–2018	151 (18.62)	52 (16.88)	
Racial or ethnic group			0.875
White	469 (57.8)	178 (57.8)	
Black	239 (29.5)	94 (30.5)	
Other/unknown	103 (12.7)	36 (11.7)	

Data are count (%) unless otherwise indicated. *P* values were obtained from t test or chi‐squared tests as appropriate. PICH indicates primary intracerebral hemorrhage; and SAH, subarachnoid hemorrhage.

* Denotes significant difference among subtypes (*P*<0.05).

An overview of changes in baseline characteristics across the study period was presented in Table 2. Over the 24 years, the mean age at HS onset remained relatively stable (*P*=0.83). However, the racial and ethnic composition of HS cases shifted significantly, marked by a decreasing proportion of White individuals and an increase in Black patients (*P*=0.001). These changes were largely reflective of demographic shifts occurring within the White population, as detailed in Table S1. Additionally, the surveillance area experienced a marked demographic transition, with the proportion of residents aged 65 years and older declining from approximately 10.15% in 1995 to 2000 to 6.21% by 2013 to 2018 (*P*<0.0001). Parallel declines were also observed in the relative proportions of female, White, and Black residents over the same time frame (*P*<0.0001).

**Table 2:** Changes in Baseline Characteristics of First Ever Hemorrhagic Stroke Over Time

	1995–2000	2001–2006	2007–2012	2013–2018	*P* value
	n=351	n=353	n=212	n=203	
Age, y (mean±SD)	63.9±16.8	61.1±17.4	63.51±17.5	60.7±16.6	0.83
Age>55 y	256 (72.9)	232 (65.7)	140 (66.0)	126 (62.0.0)	0.001*
Age, y					0.001*
<45	55 (15.7)	63 (17.85)	34 (16.0)	30 (14.8)	
45–54	40 (11.4)	58 (16.43)	38 (17.9)	47 (23.2)	
55–64	59 (16.8)	66 (18.7)	30 (14.2)	50 (24.6)	
65–74	86 (24.5)	65 (18.4)	35 (16.5)	22 (10.8)	
75–84	74 (21.08)	71 (20.1)	52 (24.5)	35 (17.2)	
85+	37 (10.54)	30 (8.5)	23 (10.9)	19 (9.4)	
Female sex	162 (46.2)	161 (45.6)	105 (49.5)	95 (46.8)	0.827
Racial or ethnic group					0.001*
White	232 (66.1)	205 (58.1)	112 (52.8)	98 (48.3)	
Black	87 (24.8)	97 (27.5)	72 (34.0)	77 (37.9)	
Other/unknown	32 (9.1)	51 (14.5)	28 (13.2)	28 (13.8)	

Data are count (%) unless otherwise indicated. *P* values were obtained from ANOVA or chi‐squared tests as appropriate.

* Denotes significance (*P*<0.05).

### Trends in HS Incidence

During 1995 to 2000, the crude annual incidence of first‐ever HS was 27.63 per 100 000 population (351 cases from 1 270 380 person‐years [95% CI, 24.81–30.68]). By 2013 to 2018, this rate had declined substantially to 10.45 per 100 000 (203 cases from 1 942 080 person‐years [95% CI, 9.06–11.99]). Comparable downward trends were observed for both PICH and SAH, although the magnitude of reduction differed across age, sex, and racial or ethnic subgroups. Table S2 presents detailed numerator and denominator data, along with crude incidence estimates stratified by demographic characteristics.

**Figure 1:**
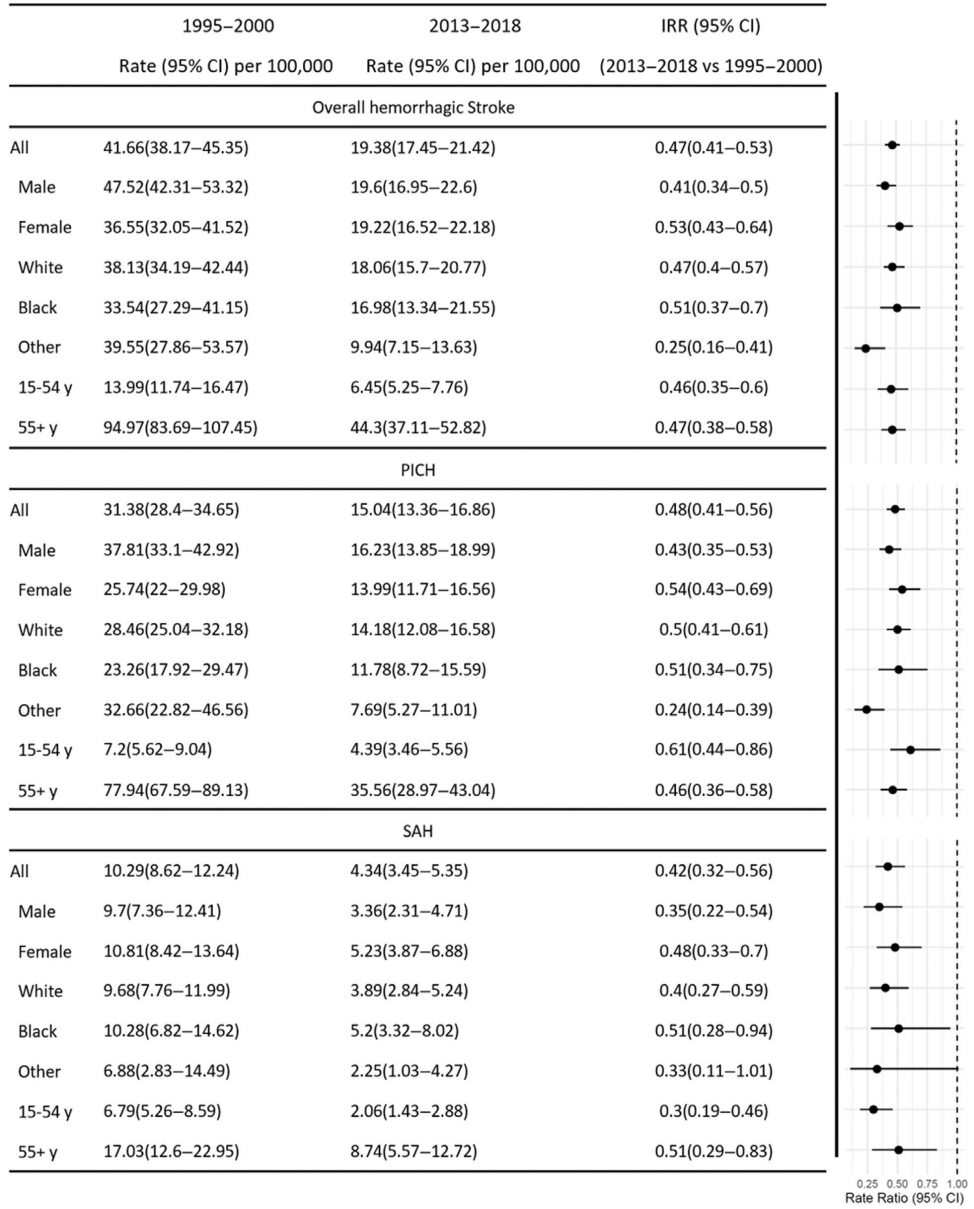
Standardized^†^ annual incidences per 100 000 per year (95% CI) of first hemorrhagic stroke over time, stratified by sex, race*, and age groups. *Other indicates races other than White or Black. ^†^To the 2011 census population of England and Wales. Complete information for other periods is available in the supplemental materials Table S3. IRR indicates incidence rate ratio; PICH, primary intracerebral hemorrhage; and SAH, subarachnoid hemorrhage.

**Figure 2:**
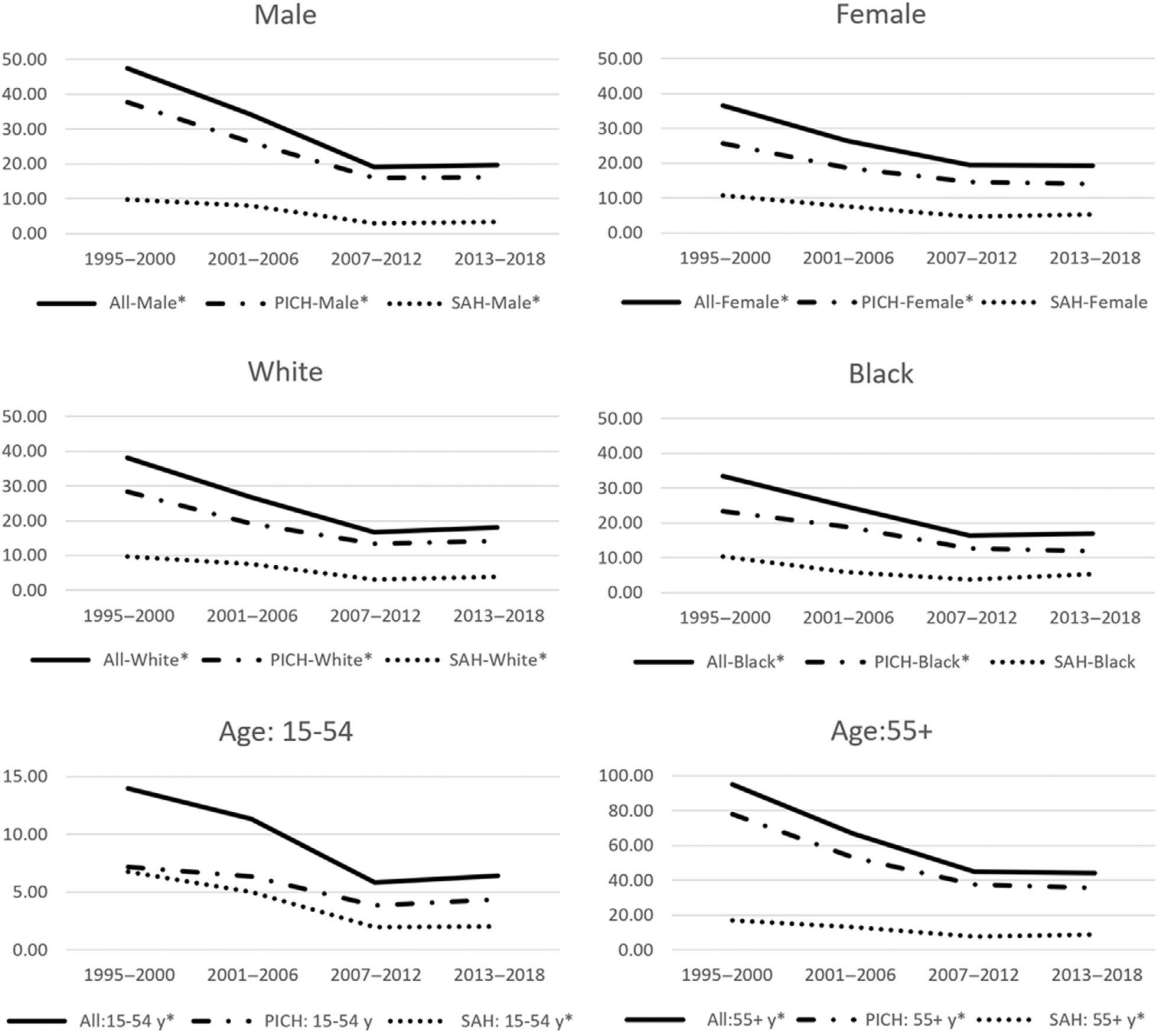
Trends in the age‐standardized† annual incidence per 100 000 per year for first‐ever hemorrhagic strokes by sex, race, and age. ^†^To the 2011 population of England and Wales. *P* values were obtained from the Cochran‐Armitage tests for trend. *denotes significant trends (*P*<0.05). PICH indicates primary intracerebral hemorrhage; and SAH, subarachnoid hemorrhage.

Figures 1 and 2 display standardized incidence rates using the 2011 England and Wales census population, with detailed estimates presented in Table S3. Between 1995 to 2000 and 2013 to 2018, the incidence of first‐ever HS declined by 53%, from 41.66 per 100 000 (529 cases from 1 270 380 person‐years) to 19.38 per 100 000 (376 cases from 1 942 080 person‐years), corresponding to an IRR of 0.47 (95% CI, 0.41–0.53). This decline was primarily driven by a significant decrease in PICH (31.38 [399/1270380]—15.04 [292/1942080], *P*<0.01).

Comparatively, women demonstrated less pronounced reductions in HS incidence than men (47% versus 59% respectively) and exhibited lower or comparable risks at all‐time points (Figure 1). This difference can be traced back to a substantial decrease in PICH and SAH incidence among men (Figure 2), showcasing a 57% reduction (*P*<0.001) in PICH and 65% in SAH (*P*<0.05), as opposed to the 46% (*P*<0.01) and 52% reductions noted in women (*P*=0.1) respectively.

Regarding racial variations, the White population experienced a 53% reduction in HS incidence between 1995 to 2000 and 2013 to 2018 (IRR, 0.47 [95% CI, 0.40–0.57]), and a 49% decline was observed among the Black population (IRR, 0.51 [95% CI, 0.37–0.70]). For PICH, comparable decreases were noted in both groups—White individuals showed an IRR of 0.50 (95% CI, 0.41–0.61) and Black individuals an IRR of 0.51 (95% CI, 0.34–0.75). The SAH incidence also fell substantially, by 60% in the White group (IRR, 0.40 [95% CI, 0.27–0.59]) and by 49% in the Black group (IRR, 0.51 [95% CI, 0.28–0.94]).

The reductions in HS incidence were similar in younger individuals aged 15 to 45 years and the older group aged 55+ (54% versus 53% respectively), despite the inherently higher risk associated with the older population. Regarding HS causes, the older population exhibited larger reductions in PICH (IRR, 0.46 [95% CI, 0.36–0.58]), whereas the young population demonstrated substantial reductions in SAH (IRR, 0.3 [95% CI, 0.19–0.46]).

### Trends in Risk Factors and Medication Use

**Figure 3:**
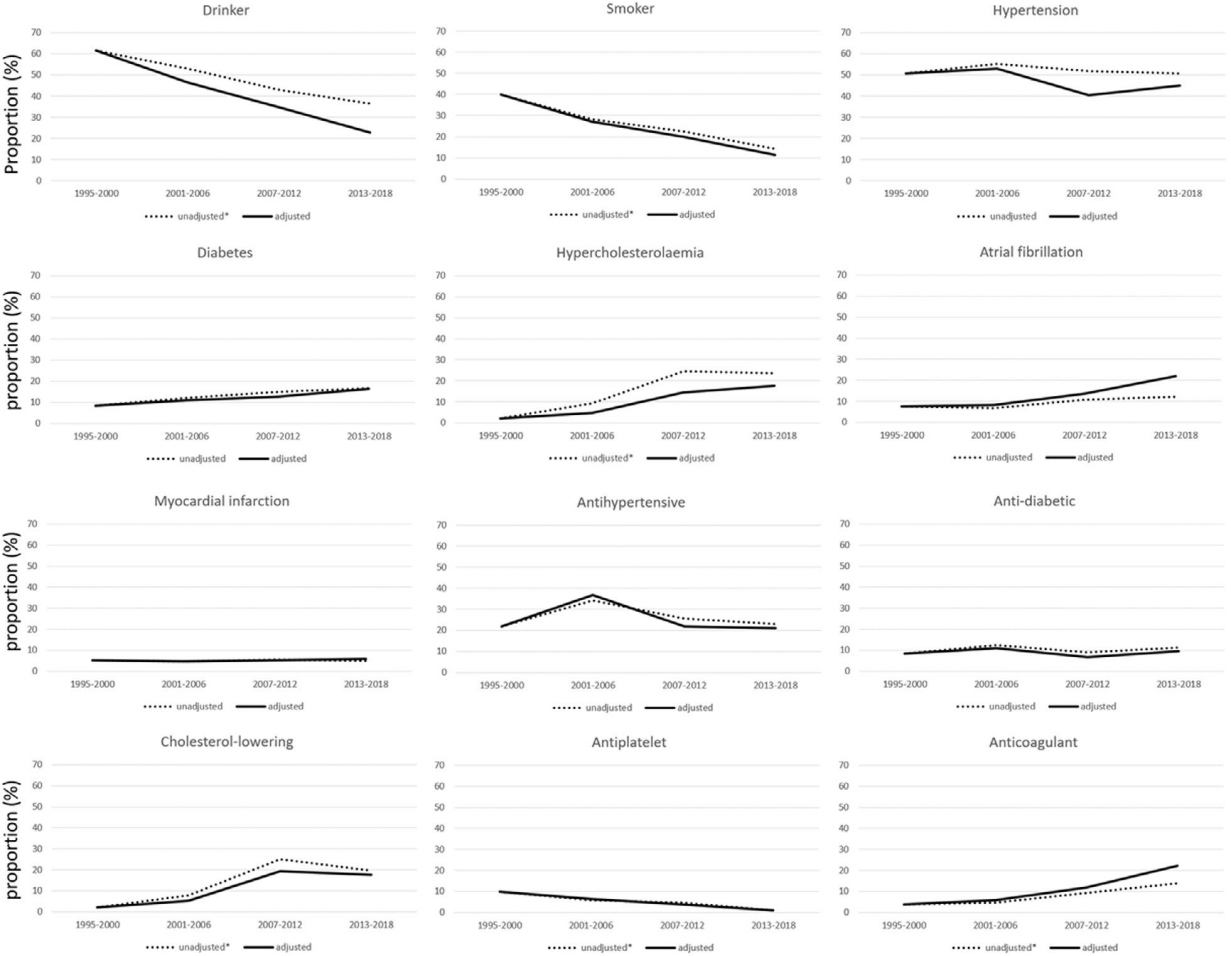
Prior risk factors and medication use over time in patients with incident first‐ever hemorrhagic stroke. *P* values were obtained by the Cochran‐Armitage tests for trend and are presented for the unadjusted rates. *denotes significant trends (*P*<0.05). ^†^Adjusted for age, sex, and race.

**Figure 4:**
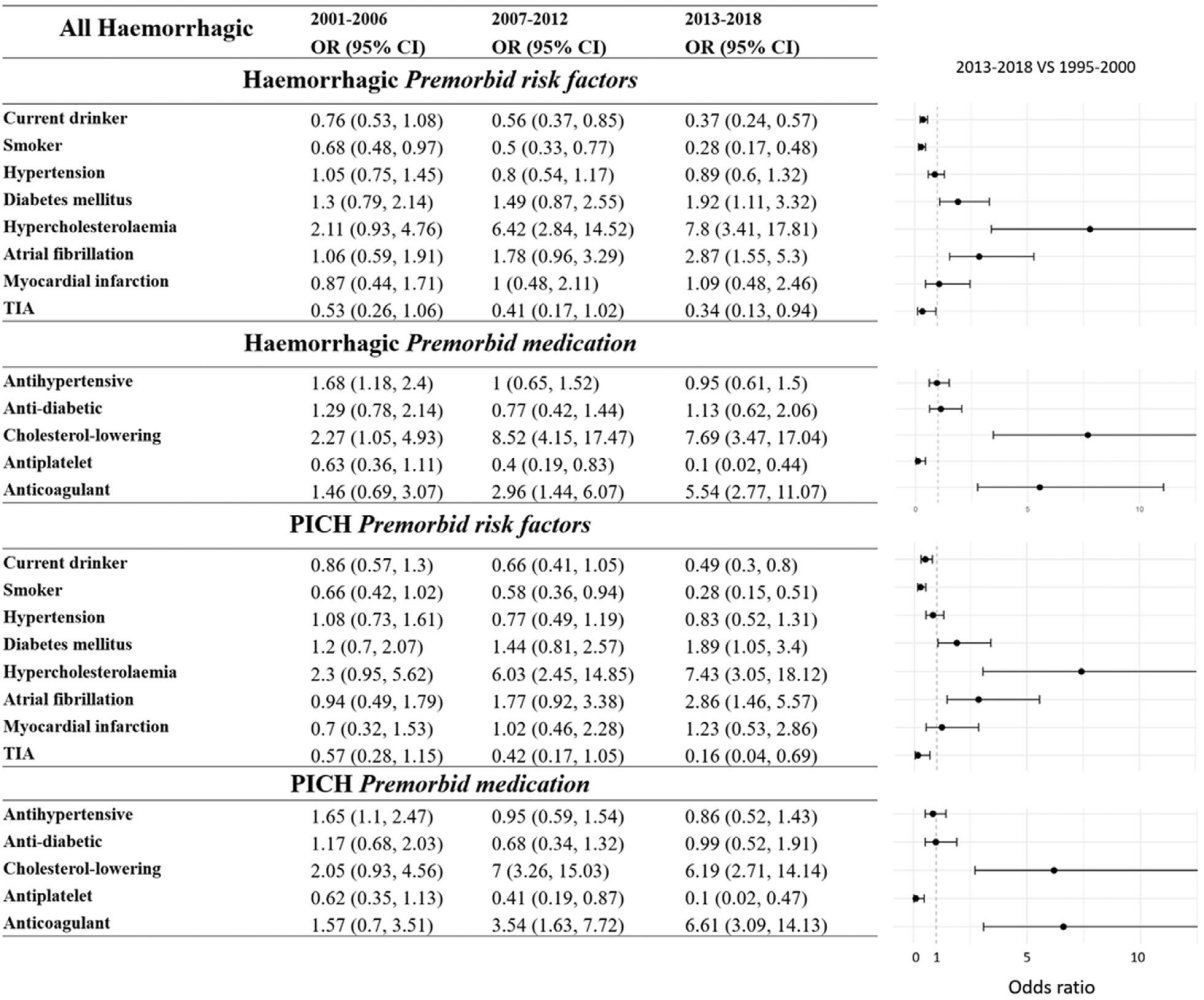
Multiply adjusted^†^ changes in risk factor profile in patients with first‐ever hemorrhagic stroke (with reference to 1995–2000 index cases). Data are OR (95% CI). ^†^For age, sex, race after multiple imputation of missing values. OR indicates odds ratio; PICH, primary intracerebral hemorrhage; and TIA, transient ischemic attack.

Figure 3 presents both unadjusted and demographically adjusted trends in the selected prestroke characteristics among patients with first‐ever HS. Figure 4 illustrates the magnitude of changes over each time period, using 1995 to 2000 as a reference, based on models multiply adjusted for age, sex, and race or ethnicity. When compared with the 1995 to 2000 cohort, the 2013 to 2018 cohort exhibited a substantial 53% reduction in the number of drinkers (OR, 0.37 [95% CI, 0.24–0.57]). Additionally, there was a significant decrease in the proportion of regular or former smokers (OR, 0.28 [95% CI, 0.17–0.48]). In contrast, the prevalence of several comorbidities increased substantially during the same period: diabetes (OR, 1.92 [95% CI, 1.11–3.32]), hypercholesterolemia (OR, 7.8 [95% CI, 3.41–17.81]), and atrial fibrillation (OR, 2.87 [95% CI, 1.55–5.30]), corresponding to relative increases of 92%, 680%, and 187%, respectively. No significant change was observed for hypertension.

Additionally, only the prophylactic use of antiplatelet drugs displayed a substantial reduction, dropping to 10% (OR, 0.10 [95% CI, 0.02–0.44]). In contrast, the premorbid use of cholesterol‐lowering medications rose sharply—approximately an 8‐fold increase after adjustment for age, sex, and race or ethnicity (OR, 7.8 [95% CI, 3.41–17.81]). A similarly significant upward trend was seen in anticoagulant use, which increased nearly 5‐fold over the 24‐year period (OR, 5.54 [95% CI, 2.77–11.07]). Similar patterns in risk factors and medication usage were found in patients with PICH, as shown in Figure 4.

Tables S4 to S22 provide detailed stratified analyses across demographic and pathogenetic subgroups. Among all patients with HS, a significant rise in diabetes prevalence was observed exclusively in women (*P*<0.01). Throughout the study period, Black individuals consistently exhibited higher rates of both hypertension and diabetes. Notably, a significant upward trend in atrial fibrillation was detected only within the Black subgroup (*P*<0.05), whereas no such trend was in White patients (*P*=0.09). Younger individuals (<55 years) reported higher rates of smoking and alcohol consumption compared with older adults. There were no substantial changes in hypertension across age subgroups. In the older group, there was a 6‐fold increase in anticoagulant usage.

## DISCUSSION

The incidence of HS decreased by 50% from 1995 to 2018 in this population‐based study of a multiracial and multiethnic population in South London. This downtrend was consistently noticeable across various demographic categories and HS subtypes (ICH and SAH). We observed an increasing trend in the prevalence of most cardiovascular risk factors, except for hypertension, which remained stable. There was a significant reduction in smoking and alcohol consumption. Additionally, the trends in the usage of antithrombotic medications illustrated diverging patterns, marked by a decrease to 10% in the uptake of antiplatelet drugs, whereas anticoagulant use saw a 5‐fold increase. However, the SAH incidence rate reduction occurred in line with the decreased premorbid smoking, which is a powerful risk factor.

### Trends in ICH and SAH Incidence

Precise temporal monitoring of the incidence of PICH and SAH remains a challenge in many studies. Our investigation provides robust evidence of a declining trend. Data from OXVASC (Oxford Vascular Study) highlighted a 37% decline in the age‐standardized incidence of ICH from 1981 to 1986 to 2002 to 2004 in a predominantly White population (94%), but the trend exists only in patients <75 years.^5^ The OXVASC study also reported no significant reduction in the incidence of SAH between 1981 to 1986 and 2002 to 2008.^31^ In contrast, a recent systematic review and meta‐analysis on aneurysmal SAH depicted a global decline in SAH incidence from 1980 to 2010, with marked regional differences that paralleled a decrease in blood pressure and smoking prevalence.^11^

Within our SLSR White cohort, a more pronounced reduction was noted: a 50% decline in ICH and a 60% decrease in SAH over a similar but more recent period (1995–2018). Factors such as advancements in public health awareness and health care services may have contributed. In addition, the SLSR area experienced rapid economic development and a significant influx of immigrants in recent decades, potentially contributing to the observed disparity.^25^, ^32^, ^33^ Unlike the OXVASC studies,^5^, ^31^ our study built upon continuous epidemiological surveillance rather than 2 distinct time periods, and the larger sample size in SLSR (n=811 versus n=107 patients with ICH in OXVASC, n=308 versus n=85 patients with SAH in OXVASC) provided sufficient power to characterize variations across 24 years. Additionally, the limited ethnic diversity in OXVASC inhibits the generalizability of its results to more ethnically diverse settings.

Likewise, age‐specific data from population‐based studies in France (1981–2006) and the Netherlands (1998–2010) demonstrated a decreased ICH incidence in patients <75 years.^4^, ^16^ However, contrasting our findings, static incidence over time in patients ≥75 years was observed in the Netherlands,^4^ whereas an increased incidence was found in the same age group in the Framingham Heart Study^17^ and Dijon.^16^ These discrepancies may result from variations in health care policy in different countries.

Our findings indicate distinct trends in HS incidence by sex and race or ethnicity over time. Specifically, SAH incidence was consistently higher in women than in men, and this sex disparity widened over time. In contrast, PICH incidence was higher in men, but the gap between the sexes has narrowed over the study period, suggesting a greater decline in PICH incidence among men compared with women.

Regarding race and ethnicity, SAH incidence was higher in Black individuals compared with White individuals, whereas the reverse trend was observed for PICH, with White individuals having higher PICH incidence than Black individuals. Notably, the greatest decline in HS incidence was observed in individuals categorized as “Other” ethnicity, and the smallest decline was seen in Black individuals. These findings suggest potential disparities in risk factor exposure, health care access, or preventive measures that needs further investigation.

Ethnic group comparisons have been seldom addressed. A US repeated cross‐sectional design study,^34^ using the Nationwide Inpatient Sample (2004–2018) and US Census Bureau data, reported an 11% increase in nationwide ICH incidence over 15 years (2004–2018), with the incidence among the Black population significantly higher than the White population. Aligned with this US study, we found that the Black population in our study had an increased risk of PICH compared with the White group (crude incidence), despite higher standardized incidence rates within the White population. Given that London's population is more diverse than that of England and Wales, the consistently recorded higher levels of hypertension and diabetes among Black patients across all SLSR time periods may provide a potential explanation, considering the established association between hypertension and ICH.^35^

### ICH Risk Factors Profile

In our study, the risk factor profile for ICH revealed a worsening trend in cardiovascular health, However, no substantial temporal shifts in the prevalence of hypertension were observed. Although arterial hypertension is a well‐established risk factor for ICH in the general population, its association with deep ICH has been more frequently reported.^35^ A meta‐analysis revealed a higher prevalence of arterial hypertension in patients with deep ICH as opposed to lobar ICH.^36^ Our findings underscore the significance of hypertension, with prevalence rates ranging between 40% and 50% across 24 years, as a common risk factor contributing to ICH. At this stage, however, we are unable to assess its specific contribution to lobar or deep ICH, which may influence the observed decline in incidence.

Between 1995 to 2000 and 2013 to 2018, we observed that the proportions of smokers and drinkers among patients with HS declined by 63% and 72%, respectively. Tobacco use is a well‐established risk factor that increases the risk of both spontaneous ICH^37^, ^38^, ^39^, ^40^ and SAH^41^ across diverse populations and ethnic groups. The relationship between alcohol consumption and stroke is more complex. A U‐shaped association has been observed between alcohol intake and ischemic stroke, whereas a more linear relationship has been found with HS, in which chronic heavy drinking increases the incidence and is associated with poorer outcomes.^41^, ^42^, ^43^, ^44^, ^45^ Therefore, the decline in smoking and alcohol use may have contributed to the observed decrease in HS incidence. However, it should be noted that the decline in these risk factors was observed among patients with stroke, not in the general population.

#### High Lipid Levels and Statin Use

The use of cholesterol‐lowering agents, mainly statins, tripled in our study population since 1995, with a consistent trend across demographic groups. Statins are well established in reducing ischemic stroke risk (~20%),^46^, ^47^ but concerns have persisted regarding a potential increase in ICH risk, based on studies such as SPARCL (Stroke Prevention by Aggressive Reduction in Cholesterol Levels).^48^, ^49^ However, a meta‐analysis of 31 randomized controlled trials found no significant increase in ICH risk with statin use.^50^ As suggested by Chaturvedi, lipid lowering may still be beneficial if combined with blood pressure and anticoagulation control.^51^ Thus, the impact of increased statin use on ICH incidence remains inconclusive.

In contrast, observational and genetic evidence, including the China Kadoorie Biobank study^52^ and a meta‐analysis of 23 prospective studies,^53^ indicates that higher low‐density lipoprotein cholesterol and total cholesterol levels are associated with a lower risk of HS. Therefore, the observed decline in ICH incidence in our study may partly reflect rising premorbid lipid levels.

#### Anticoagulant Usage and Promorbid Atrial Fibrillation

We observed a steady increase in anticoagulant use over the 24‐year period, with a sharper rise from 2007 to 2018. Oral anticoagulants (OACs), particularly warfarin, have been associated with increased hematoma volume, hematoma growth, and ICH‐related mortality.^16^, ^17^, ^54^, ^55^, ^56^ Population‐based studies from France and Canada in 2013 reported that 15% to 21% of ICH cases were associated with anticoagulation, mostly in individuals >65.^16^, ^56^

Although vitamin K antagonists were the predominant OACs until 2010, the uptake of direct OACs has since grown rapidly, surpassing vitamin K antagonists in some countries.^57^ Randomized trials suggest direct OACs carry a lower risk of intracranial bleeding compared with warfarin.^58^ Nevertheless, data from the Swedish stroke register show rising OAC‐associated ICH alongside declining non‐OAC‐associated ICH.^59^ Although our study did not distinguish between vitamin K antagonists and direct OACs, the increased use of anticoagulants may help explain the plateau in ICH incidence since 2007, following a marked decline from 1995 to 2006. This trend is consistent with Danish registry data.^60^

We also observed a rise in atrial fibrillation, likely reflecting improved cardiovascular survival and enhanced detection, which may have contributed to the increased use of premorbid OACs.^17^, ^61^ Although OACs and antiplatelets provide substantial vascular protection, they also increase ICH risk.^62^, ^63^

Despite this, our study found a 52% reduction in ICH incidence, suggesting additional factors may be contributing. These may include improvements in hypertension control, reductions in resistant hypertension, and unmeasured influences such as physical activity, stress, medication adherence, and genetic or environmental factors.^1^, ^39^, ^64^, ^65^, ^66^, ^67^

### Strength and Limitations

Our investigation stands as the largest population‐based study examining trends in HS incidence and risk factors, offering comprehensive insights into pathogenetic subtypes, including ICH and SAH, further classified by age, sex, race, and ethnicity. Although we pursued rigorous consistency and precision in our notification system, we acknowledge an incremental increase in case ascertainment completeness over the years (from 75% in 2001–2002 to 88% in 2005–2006).^18^, ^25^ Despite this increase not being statistically significant, any ensuing bias might intensify the sensitivity to detect HS cases in recent years, potentially countering an observed declining trend. Another strength of our study lies in the continuous, extended period of stroke patient identification in the multiethnic region of south London; the substantial sample size accumulated over 24 years provided sufficient power to examine extended trends across demographic subgroups. However, some subgroup‐specific analyses, particularly those involving subgroups with SAH and younger individuals, were limited by the statistical power due to the small numbers.

Nonetheless, our study presents several limitations. First, we could not examine the impacts of potential valuable risk factors such as hypertension on ICH trends. Second, we were unable to associate the change in incidence with a potential shift in ICH according to location. Third, the frequency of missing values was higher in earlier years, which could potentially bias the longitudinal risk factor trends. On average, 13.89% of the examined predictors were missing, with a range of 7.33% to 25.02%. Despite this, nonresponse appeared random, without an identifiable pattern, and we employed multiple imputation analysis to minimize any resultant bias. In addition, the small sample size of patients with SAH restricted our ability to adjust demographic subgroup risk factor trends. We also lack data on the classification between aneurysmal and nonaneurysmal (including angionegative) SAH, and their different causes may lead to varying incidence trends and risk factor profiles. The observed decline in SAH incidence may be confounded by the increased detection and elective treatment of unruptured aneurysms due to advances in imaging techniques. Future studies with comprehensive imaging data are needed to better assess these factors.

Lastly, we should acknowledge that the overall cardiovascular health described by the related risk factor profiles in our study cannot be directly generalized to the general population. This is due to potential inherent differences between patients with ICH and the general population. Because the SLSR is a stroke register recruiting only patients with stroke, we are not able to report risk factors and medication use for individuals who did not experience a stroke. Additionally, the risk factor profiles in the SLSR data are limited in their ability to evaluate overall cardiovascular health, as they may be influenced by factors not captured in our study, such as lifestyle, policy implementation, and health service quality.

## CONCLUSIONS

In summary, our findings highlight >50% reductions in the incidence of ICH and SAH in London, United Kingdom, across various demographic groups over a period of 24 years from 1995 to 2018. This study cautiously suggests that prevention, or at the very least, delay in the onset of some strokes may be achievable through improved risk factor management. It also underscores the importance of targeted prevention strategies to bolster the current trends and prevent future increases.

## Sources of Funding

The project is funded by the National Institute for Health and Care Research (NIHR) under its Programme Grants for Applied Research (NIHR202339). Authors Lu Liu (CSC No. 202108310074) and Xianqi Li (CSC No. 201808320212) received financial support from the China Scholarship Council‐PhD Scholarship.

## Disclosure

None.

## Supporting information

Tables S1–S22

STROBE checklist
