# Supplementary material for: Long‐Term Trends in Incidence and Risk Factors for Hemorrhagic Stroke Subtypes Over 24 Years: The South London Stroke Register
Source: J Am Heart Assoc. 2025 Aug 12;14(16):e040371. doi: 10.1161/JAHA.124.040371 (PMC12533615; doi:10.1161/JAHA.124.040371)
Supplement: Supplementary file 1 — Tables S1–S22 [file JAH3-14-e040371-s001.pdf]

Supplemental Material

Demographic changes in the SLSR population

Table S1: Changes in demographic composition of the SLSR population over time.

	1995-2000 (n=1556706)	2001-2006 (n=2242770)	2007-2012 (n=2329968)	2013-2018 (n=2280336)	P-value
**Age**					
<45	1150074 (73.88)	1679418 (74.88)	1733808 (74.41)	1697094 (74.42)	<0.0001*
45-54	139314 (8.95)	225750 (10.07)	269310 (11.56)	293034 (12.85)	<0.0001*
55-64	109260 (7.02)	143016 (6.38)	152274 (6.54)	148704 (6.52)	<0.0001*
65-74	88404 (5.68)	105354 (4.7)	94986 (4.08)	76902 (3.37)	<0.0001*
75-84	54168 (3.48)	67164 (2.99)	58746 (2.52)	46500 (2.04)	<0.0001*
85+	15486 (0.99)	22068 (0.98)	20844 (0.89)	18102 (0.79)	<0.0001*
**Age 65+ y**	158058 (10.15)	194586 (8.68)	174576 (7.49)	141504 (6.21)	<0.0001*
**Female**	789492 (50.72)	1128000 (50.29)	1162398 (49.89)	1125672 (49.36)	<0.0001*
**Ethnicity**					
White	1018038 (65.4)	1352844 (60.32)	1327824 (56.99)	1224714 (53.71)	<0.0001*
Black	407376 (26.17)	620142 (27.65)	600864 (25.79)	552516 (24.23)	<0.0001*
Other/unknown	131292 (8.43)	269784 (12.03)	401280 (17.22)	503106 (22.06)	<0.0001*

Data are count (%). P-values were obtained from the Cochran-Armitage tests for trend. * denotes significance ($p<0.05$).

Crude incidence rates

Table S2: Crude annual incidences per 100,000 per year (95% CI) of first haemorrhagic stroke over time, stratified by stroke subtypes and age, sex, and ethnicity.

	1995-2000			2001-2006			2007-2012			2013-2018			P-value for trend	IRR (95% CI)
	n	person-years	Rate (95% CI) per 100,000	n	person-years	Rate (95% CI) per 100,000	n	person-years	Rate (95% CI) per 100,000	n	person-years	Rate (95% CI) per 100,000		(2013-18 vs 1995-2000)
Haemorrhagic Stroke														
**All**	351	1270380	27.63(24.81-30.68)	353	1840206	19.18(17.23-21.29)	212	1947654	10.88(9.47-12.45)	203	1942080	10.45(9.06-11.99)	0.001186*	0.38(0.32-0.45)
**Sex**														
Male	189	622134	30.38(26.2-35.03)	192	910926	21.08(18.2-24.28)	107	973806	10.99(9-13.28)	108	983430	10.98(9.01-13.26)	0.0003648*	0.36(0.28-0.46)
Female	162	648246	24.99(21.29-29.15)	161	929280	17.33(14.75-20.22)	105	973848	10.78(8.82-13.05)	95	958650	9.91(8.02-12.11)	0.003522*	0.4(0.3-0.51)
**Ethnicity**														
White	232	885948	26.19(22.93-29.78)	205	1191372	17.21(14.93-19.73)	112	1193328	9.39(7.73-11.29)	98	1126854	8.7(7.06-10.6)	0.0005846*	0.33(0.26-0.42)
Black	87	284904	30.54(24.46-37.67)	97	442116	21.94(17.79-26.76)	72	439620	16.38(12.81-20.63)	77	415530	18.53(14.62-23.16)	0.04661*	0.61(0.44-0.83)
Other	32	99528	32.15(21.99-45.39)	51	206718	24.67(18.37-32.44)	28	314706	8.9(5.91-12.86)	28	399696	7.01(4.65-10.12)	1.732E-06*	0.22(0.13-0.37)
**Age groups**														
15-54 y	104	1003062	10.37(8.47-12.56)	131	1502604	8.72(7.29-10.35)	75	1620804	4.63(3.64-5.8)	83	1651872	5.02(4-6.23)	0.09293	0.48(0.36-0.65)
55+ y	247	267318	92.4(81.23-104.67)	222	337602	65.76(57.39-75)	137	326850	41.92(35.19-49.55)	120	290208	41.35(34.28-49.44)	3.476E-07*	0.45(0.36-0.56)
**Age**														
15-44	56	863748	6.48(4.9-8.42)	64	1276854	5.01(3.86-6.4)	39	1351494	2.89(2.05-3.94)	31	1358838	2.28(1.55-3.24)	0.1068	0.35(0.22-0.56)
45-54	48	139314	34.45(25.4-45.68)	67	225750	29.68(23-37.69)	36	269310	13.37(9.36-18.51)	52	293034	17.75(13.25-23.27)	0.002339*	0.52(0.34-0.78)
55-64	59	109260	54(41.11-69.66)	58	143016	40.55(30.8-52.43)	28	152274	18.39(12.22-26.58)	46	148704	30.93(22.65-41.26)	0.0006561*	0.57(0.38-0.86)
65-74	84	88404	95.02(75.79-117.64)	72	105354	68.34(53.47-86.06)	37	94986	38.95(27.43-53.69)	25	76902	32.51(21.04-47.99)	2.412E-10*	0.34(0.21-0.54)
75-84	72	54168	132.92(104-167.39)	67	67164	99.76(77.31-126.69)	51	58746	86.81(64.64-114.14)	32	46500	68.82(47.07-97.15)	3.157E-06*	0.52(0.33-0.8)
85+	32	15486	206.64(141.34-291.71)	25	22068	113.29(73.31-167.23)	21	20844	100.75(62.36-154)	17	18102	93.91(54.71-150.36)	4.552E-12*	0.45(0.24-0.84)
PICH														
**All**	251	1270380	19.76(17.39-22.36)	246	1840206	13.37(11.75-15.15)	163	1947654	8.37(7.13-9.76)	151	1942080	7.78(6.58-9.12)	0.0091*	0.39(0.32-0.48)
**Sex**														
Male	144	622134	23.15(19.52-27.25)	141	910926	15.48(13.03-18.25)	89	973806	9.14(7.34-11.25)	85	983430	8.64(6.9-10.69)	0.002981*	0.37(0.28-0.49)
Female	107	648246	16.51(13.53-19.95)	105	929280	11.3(9.24-13.68)	74	973848	7.6(5.97-9.54)	66	958650	6.88(5.32-8.76)	0.02501*	0.42(0.3-0.57)
**Ethnicity**														
White	170	885948	19.19(16.41-22.3)	138	1191372	11.58(9.73-13.69)	87	1193328	7.29(5.84-8.99)	74	1126854	6.57(5.16-8.24)	0.004776*	0.34(0.26-0.45)
Black	55	284904	19.3(14.54-25.13)	72	442116	16.29(12.74-20.51)	56	439620	12.74(9.62-16.54)	56	415530	13.48(10.18-17.5)	0.232	0.7(0.47-1.03)
Other	26	99528	26.12(17.06-38.28)	36	206718	17.42(12.2-24.11)	20	314706	6.36(3.88-9.81)	21	399696	5.25(3.25-8.03)	9.134E-06*	0.2(0.11-0.37)
**Age groups**														

15-54 y	49	1003062	4.89(3.61-6.46)	70	1502604	4.66(3.63-5.89)	49	1620804	3.02(2.24-4)	56	1651872	3.39(2.56-4.4)	0.4919	0.69(0.46-1.04)
55+ y	202	267318	75.57(65.5-86.74)	176	337602	52.13(44.71-60.43)	114	326850	34.88(28.77-41.9)	95	290208	32.74(26.48-40.02)	3.09E-06*	0.43(0.34-0.56)
**Age**														
15-44	21	863748	2.43(1.5-3.72)	32	1276854	2.51(1.71-3.54)	22	1351494	1.63(1.02-2.46)	18	1358838	1.32(0.79-1.78)	0.5027	0.54(0.27-1.07)
45-54	28	139314	20.1(13.36-29.05)	38	225750	16.83(11.91-23.1)	27	269310	10.03(6.61-14.59)	38	293034	12.97(9.18-17.8)	0.1034	0.65(0.39-1.09)
55-64	46	109260	42.1(30.82-56.16)	35	143016	24.47(17.05-34.04)	26	152274	17.07(11.15-25.02)	33	148704	22.19(15.28-31.17)	0.003515*	0.53(0.33-0.84)
65-74	64	88404	72.39(55.75-92.45)	58	105354	55.05(41.8-71.17)	28	94986	29.48(19.59-42.6)	19	76902	24.71(14.88-38.58)	2.189E-08*	0.34(0.19-0.58)
75-84	65	54168	120(92.61-152.95)	58	67164	86.36(65.57-111.63)	41	58746	69.79(50.08-94.68)	27	46500	58.06(38.26-84.48)	7.305E-07*	0.48(0.3-0.77)
85+	27	15486	174.35(114.9-253.67)	25	22068	113.29(73.31-167.23)	19	20844	91.15(54.88-142.35)	16	18102	88.39(50.52-143.54)	6.745E-09*	0.51(0.26-0.98)
SAH														
**All**	100	1270380	7.87(6.4-9.57)	107	1840206	5.81(4.77-7.03)	49	1947654	2.52(1.86-3.33)	52	1942080	2.68(2-3.51)	0.05224*	0.34(0.24-0.48)
**Sex**														
Male	45	622134	7.23(5.28-9.68)	51	910926	5.6(4.17-7.36)	18	973806	1.85(1.1-2.92)	23	983430	2.34(1.48-3.51)	0.04585*	0.32(0.19-0.55)
Female	55	648246	8.48(6.39-11.04)	56	929280	6.03(4.55-7.83)	31	973848	3.18(2.16-4.52)	29	958650	3.03(2.03-4.34)	0.05924	0.36(0.22-0.57)
**Ethnicity**														
White	62	885948	7(5.37-8.97)	67	1191372	5.62(4.36-7.14)	25	1193328	2.09(1.36-3.09)	24	1126854	2.13(1.36-3.17)	0.04805*	0.3(0.18-0.49)
Black	32	284904	11.23(7.68-15.86)	25	442116	5.65(3.66-8.35)	16	439620	3.64(2.08-5.91)	21	415530	5.05(3.13-7.73)	0.06914	0.45(0.25-0.8)
Other	6	99528	6.03(2.21-13.12)	15	206718	7.26(4.06-11.97)	8	314706	2.54(1.1-5.01)	7	399696	1.75(0.7-3.61)	0.06107*	0.29(0.08-1.08)
**Age groups**														
15-54 y	55	1003062	5.48(4.13-7.14)	61	1502604	4.06(3.11-5.21)	26	1620804	1.6(1.05-2.35)	27	1651872	1.63(1.08-2.38)	0.07954	0.3(0.18-0.48)
55+ y	45	267318	16.83(12.28-22.53)	46	337602	13.63(9.98-18.17)	23	326850	7.04(4.46-10.56)	25	290208	8.61(5.57-12.72)	0.03957*	0.51(0.3-0.85)
**Age**														
15-44	35	863748	4.05(2.82-5.64)	32	1276854	2.51(1.71-3.54)	17	1351494	1.26(0.73-2.01)	13	1358838	0.96(0.51-1.64)	0.1123	0.24(0.11-0.46)
45-54	20	139314	14.36(8.77-22.17)	29	225750	12.85(8.6-18.45)	9	269310	3.34(1.53-6.34)	14	293034	4.78(2.61-8.02)	0.004002*	0.33(0.16-0.69)
55-64	13	109260	11.9(6.34-20.35)	23	143016	16.08(10.19-24.13)	2	152274	1.31(0.16-4.74)	13	148704	8.74(4.65-14.95)	0.07863	0.73(0.31-1.72)
65-74	20	88404	22.62(13.82-34.94)	14	105354	13.29(7.26-22.3)	9	94986	9.48(4.33-17.99)	6	76902	7.8(2.86-16.98)	0.003075*	0.34(0.11-0.89)
75-84	7	54168	12.92(5.2-26.63)	9	67164	13.4(6.13-25.44)	10	58746	17.02(8.16-31.3)	5	46500	10.75(3.49-25.09)	0.8605	0.83(0.21-3.05)
85+	5	15486	32.29(10.48-75.35)	0	22068	0(0-16.72)	2	20844	9.6(1.16-34.66)	1	18102	5.52(0.14-30.78)	4.37E-06*	0.17(0.0036-1.53)

n indicates number of cases; CI, confidence interval; and IRR, incidence rate ratio.

P-values were obtained from the Cochran-Armitage tests for trend after correcting for multiple comparisons.* denotes significant trends ($p<0.05$).

Standardised incidence rates to census population of England & Wales (2011) – EW11

Table S3: Age-adjusted trends in annual incidence per 100,000 per year (95% CI).

	1995-2000		2001-2006		2007-2012		2013-2018		P-value for trend	IRR (95% CI)
	n / N	Rate (95% CI) per 100,000	n / N	Rate (95% CI) per 100,000	n / N	Rate (95% CI) per 100,000	n / N	Rate (95% CI) per 100,000		(2013-18 vs 1995-2000)
Haemorrhagic Stroke										
**All**	529/1270380	41.66(38.17-45.35)	556/1840206	30.23(27.75-32.83)	375/1947654	19.26(17.35-21.3)	376/1942080	19.38(17.45-21.42)	<0.0001*	0.47(0.41-0.53)
**Sex**										
Male	296/622134	47.52(42.31-53.32)	313/910926	34.34(30.66-38.39)	186/973806	19.11(16.45-22.05)	193/983430	19.6(16.95-22.6)	<0.0001*	0.41(0.34-0.5)
Female	237/648246	36.55(32.05-41.52)	246/929280	26.47(23.27-30)	189/973848	19.41(16.74-22.38)	184/958650	19.22(16.52-22.18)	<0.01*	0.53(0.43-0.64)
**Ethnicity**										
White	338/885948	38.13(34.19-42.44)	318/1191372	26.72(23.84-29.79)	198/1193328	16.62(14.36-19.07)	204/1126854	18.06(15.7-20.77)	<0.01*	0.47(0.4-0.57)
Black	96/284904	33.54(27.29-41.15)	109/442116	24.64(20.24-29.74)	72/439620	16.32(12.81-20.63)	71/415530	16.98(13.34-21.55)	<0.01*	0.51(0.37-0.7)
Other	39/99528	39.55(27.86-53.57)	59/206718	28.48(21.73-36.82)	38/314706	11.98(8.54-16.57)	40/399696	9.94(7.15-13.63)	<0.0001*	0.25(0.16-0.41)
**Age**										
15-54 y	140/1003062	13.99(11.74-16.47)	170/1502604	11.34(9.68-13.15)	95/1620804	5.85(4.74-7.17)	106/1651872	6.45(5.25-7.76)	<0.05*	0.46(0.35-0.6)
55+ y	254/267318	94.97(83.69-107.45)	225/337602	66.61(58.22-75.95)	147/326850	45.09(38-52.86)	129/290208	44.3(37.11-52.82)	<0.0001*	0.47(0.38-0.58)
PICH										
**All**	399/1270380	31.38(28.4-34.65)	412/1840206	22.4(20.28-24.66)	299/1947654	15.35(13.66-17.19)	292/1942080	15.04(13.36-16.86)	<0.01*	0.48(0.41-0.56)
**Sex**										
Male	235/622134	37.81(33.1-42.92)	240/910926	26.38(23.12-29.9)	156/973806	16.05(13.6-18.74)	160/983430	16.23(13.85-18.99)	<0.001*	0.43(0.35-0.53)
Female	167/648246	25.74(22-29.98)	174/929280	18.76(16.05-21.72)	143/973848	14.64(12.38-17.3)	134/958650	13.99(11.71-16.56)	<0.05*	0.54(0.43-0.69)
**Ethnicity**										
White	252/885948	28.46(25.04-32.18)	229/1191372	19.24(16.81-21.88)	161/1193328	13.48(11.49-15.74)	160/1126854	14.18(12.08-16.58)	<0.05*	0.5(0.41-0.61)
Black	66/284904	23.26(17.92-29.47)	83/442116	18.88(14.95-23.27)	56/439620	12.67(9.62-16.54)	49/415530	11.78(8.72-15.59)	<0.05*	0.51(0.34-0.75)
Other	33/99528	32.66(22.82-46.56)	41/206718	19.66(14.23-26.91)	27/314706	8.58(5.65-12.48)	31/399696	7.69(5.27-11.01)	<0.0001*	0.24(0.14-0.39)
**Age**										
15-54 y	72/1003062	7.2(5.62-9.04)	95/1502604	6.34(5.12-7.73)	63/1620804	3.87(2.99-4.97)	73/1651872	4.39(3.46-5.56)	0.297	0.61(0.44-0.86)
55+ y	208/267318	77.94(67.59-89.13)	180/337602	53.34(45.81-61.7)	122/326850	37.45(31-44.57)	103/290208	35.56(28.97-43.04)	<0.0001*	0.46(0.36-0.58)
SAH										
**All**	131/1270380	10.29(8.62-12.24)	144/1840206	7.82(6.6-9.21)	76/1947654	3.91(3.07-4.88)	84/1942080	4.34(3.45-5.35)	0.058	0.42(0.32-0.56)
**Sex**										
Male	60/622134	9.7(7.36-12.41)	73/910926	7.96(6.28-10.08)	30/973806	3.06(2.08-4.4)	33/983430	3.36(2.31-4.71)	<0.05*	0.35(0.22-0.54)
Female	70/648246	10.81(8.42-13.64)	72/929280	7.71(6.06-9.76)	46/973848	4.77(3.46-6.3)	50/958650	5.23(3.87-6.88)	0.099	0.48(0.33-0.7)

**Ethnicity**										
White	86/885948	9.68(7.76-11.99)	89/1191372	7.49(6-9.19)	38/1193328	3.14(2.25-4.37)	44/1126854	3.89(2.84-5.24)	<0.05*	0.4(0.27-0.59)
Black	29/284904	10.28(6.82-14.62)	25/442116	5.76(3.66-8.35)	16/439620	3.65(2.08-5.91)	22/415530	5.2(3.32-8.02)	0.12	0.51(0.28-0.94)
Other	7/99528	6.88(2.83-14.49)	18/206718	8.82(5.16-13.76)	11/314706	3.4(1.74-6.25)	9/399696	2.25(1.03-4.27)	0.062	0.33(0.11-1.01)
**Age**										
15-54 y	68/1003062	6.79(5.26-8.59)	75/1502604	5(3.93-6.26)	32/1620804	1.97(1.35-2.79)	34/1651872	2.06(1.43-2.88)	0.05284*	0.3(0.19-0.46)
55+ y	46/267318	17.03(12.6-22.95)	45/337602	13.27(9.72-17.84)	25/326850	7.64(4.95-11.29)	25/290208	8.74(5.57-12.72)	0.04588*	0.51(0.29-0.83)

n indicates number of cases; N, number at risk; CI, confidence interval; and IRR, incidence rate ratio.

P-values were obtained from the Cochran-Armitage tests for trend after correcting for multiple comparisons.

* denotes significant trends ($p<0.05$).

Table S4 Trends in risk factors in overall HS male patients

Male	**1995-2000**	**2001-2006**	**2007-2012**	**2013-2018**	P-value (trend)†	Adjusted model‡ OR (95% CI)
Premorbid risk factors						
**Current drinker**	133 (70.4)	117 (60.9)	55 (51.4)	52 (48.1)	0.0005625*	0.37(0.2, 0.67)
**Smoker**	91 (48.1)	64 (33.3)	31 (29)	17 (15.7)	1.017e-06*	0.24(0.12, 0.47)
**Hypertension**	93 (49.2)	98 (51)	56 (52.3)	56 (51.9)	0.68	1.01(0.6, 1.7)
**Diabetes mellitus**	19 (10.1)	27 (14.1)	15 (14)	16 (14.8)	0.3474	1.51(0.72, 3.19)
**Hypercholesterolaemia**	4 (2.1)	20 (10.4)	30 (28)	24 (22.2)	1.674e-06*	8.78(2.91, 26.55)
**Atrial fibrillation**	14 (7.4)	13 (6.8)	9 (8.4)	15 (13.9)	0.1016	3.86(1.66, 8.99)
**Myocardial infarction**	12 (6.3)	10 (5.2)	9 (8.4)	6 (5.6)	0.9395	1.05(0.36, 3.04)
**TIA**	11 (5.8)	8 (4.2)	4 (3.7)	5 (4.6)	0.6691	0.76(0.25, 2.34)
Premorbid medication						
**Antihypertensive**	39 (20.6)	62 (32.3)	32 (29.9)	27 (25)	0.5887	1.19(0.66, 2.16)
**Anti-diabetic**	19 (10.1)	27 (14.1)	11 (10.3)	11 (10.2)	0.8113	0.91(0.4, 2.07)
**Cholesterol-lowering**	5 (2.6)	16 (8.3)	31 (29)	22 (20.4)	3.999e-06*	7.54(2.84, 20.03)
**Antiplatelet**	14 (7.4)	14 (7.3)	6 (5.6)	1 (0.9)	0.0352*	0.14(0.02, 1.1)
**Anticoagulant**	10 (5.3)	6 (3.1)	11 (10.3)	15 (13.9)	0.007075*	4.96(2.03, 12.12)

OR indicates odds ratio; CI, confidence interval; and TIA, transient ischaemic attack. † Cochran-Armitage tests for trend. ‡ 2013-2018 with reference to 1995-2000 after adjusting for age and ethnicity

Table S5 Trends in risk factors in overall HS female patients

Female	**1995-2000**	**2001-2006**	**2007-2012**	**2013-2018**	P-value (trend)†	Adjusted model‡ OR (95% CI)
Premorbid risk factors						
**Current drinker**	83 (51.2)	70 (43.5)	36 (34.3)	22 (23.2)	*1.694e-05**	0.35(0.19, 0.66)
**Smoker**	49 (30.2)	36 (22.4)	17 (16.2)	12 (12.6)	0.001045*	0.36(0.17, 0.75)
**Hypertension**	85 (52.5)	97 (60.2)	54 (51.4)	47 (49.5)	0.4244	0.75(0.41, 1.37)
**Diabetes mellitus**	11 (6.8)	16 (9.9)	17 (16.2)	18 (18.9)	0.004453*	2.61(1.13, 6.05)
**Hypercholesterolaemia**	4 (2.5)	13 (8.1)	22 (21)	24 (25.3)	1.92e-07*	7.31(2.35, 22.75)
**Atrial fibrillation**	13 (8)	11 (6.8)	14 (13.3)	10 (10.5)	0.2886	2.11(0.82, 5.44)
**Myocardial infarction**	7 (4.3)	7 (4.3)	3 (2.9)	4 (4.2)	0.8342	1.16(0.32, 4.24)
**TIA**	13 (8)	6 (3.7)	3 (2.9)	0 (0)	0.002962*	N/A
Premorbid medication						
**Antihypertensive**	38 (23.5)	59 (36.6)	22 (21)	20 (21.1)	0.2396	0.72(0.37, 1.41)
**Anti-diabetic**	11 (6.8)	17 (10.6)	8 (7.6)	12 (12.6)	0.2639	1.54(0.62, 3.8)
**Cholesterol-lowering**	3 (1.9)	12 (7.5)	22 (21)	18 (18.9)	1.026e-05*	8.18(2.3, 29.11)
**Antiplatelet**	21 (13)	7 (4.3)	4 (3.8)	1 (1.1)	0.0003933*	0.07(0.01, 0.55)
**Anticoagulant**	4 (2.5)	11 (6.8)	9 (8.6)	13 (13.7)	0.003344*	7.45(2.31, 24.04)

OR indicates odds ratio; CI, confidence interval; and TIA, transient ischaemic attack. † Cochran-Armitage tests for trend. ‡ 2013-2018 with reference to 1995-2000 after adjusting for age and ethnicity

Table S6 Trends in risk factors in overall HS white patients

White	1995-2000	2001-2006	2007-2012	2013-2018	P-value (trend)†	Adjusted model‡ OR (95% CI)
Premorbid risk factors						
**Current drinker**	149 (64.2)	115 (56.1)	49 (43.8)	45 (45.9)	0.002602*	0.41(0.22, 0.74)
**Smoker**	99 (42.7)	62 (30.2)	28 (25)	13 (13.3)	3.102e-06*	0.2(0.1, 0.42)
**Hypertension**	117 (50.4)	104 (50.7)	56 (50)	35 (35.7)	0.04423*	0.59(0.35, 1.01)
**Diabetes mellitus**	13 (5.6)	16 (7.8)	13 (11.6)	11 (11.2)	0.1073	2.28(0.98, 5.35)
**Hypercholesterolaemia**	3 (1.3)	17 (8.3)	30 (26.8)	17 (17.3)	1.227e-05*	6.97(2.11, 23.01)
**Atrial fibrillation**	25 (10.8)	19 (9.3)	21 (18.8)	16 (16.3)	0.09008	2.15(1.05, 4.39)
**Myocardial infarction**	16 (6.9)	11 (5.4)	9 (8)	4 (4.1)	0.5885	0.66(0.21, 2.08)
**TIA**	17 (7.3)	7 (3.4)	7 (6.3)	3 (3.1)	0.3069	0.4(0.11, 1.42)
Premorbid medication						
**Antihypertensive**	47 (20.3)	59 (28.8)	21 (18.8)	13 (13.3)	0.08463	0.67(0.35, 1.31)
**Anti-diabetic**	13 (5.6)	17 (8.3)	5 (4.5)	7 (7.1)	0.9423	1.45(0.55, 3.82)
**Cholesterol-lowering**	4 (1.7)	15 (7.3)	31 (27.7)	15 (15.3)	4.834e-05*	6.39(2.34, 17.43)
**Antiplatelet**	31 (13.4)	10 (4.9)	7 (6.3)	1 (1)	0.001105*	0.07(0.01, 0.55)
**Anticoagulant**	13 (5.6)	13 (6.3)	16 (14.3)	14 (14.3)	0.0117*	3.79(1.66, 8.66)

OR indicates odds ratio; CI, confidence interval; and TIA, transient ischaemic attack. † Cochran-Armitage tests for trend. ‡ 2013-2018 with reference to 1995-2000 after adjusting for age and ethnicity

Table S7 Trends in risk factors in overall HS black patients

Black	**1995-2000**	**2001-2006**	**2007-2012**	**2013-2018**	P-value (trend)†	Adjusted model‡ OR (95% CI)
Premorbid risk factors						
**Current drinker**	54 (62.1)	49 (50.5)	35 (48.6)	22 (28.6)	4.52e-06*	0.29(0.13, 0.61)
**Smoker**	30 (34.5)	23 (23.7)	18 (25)	9 (11.7)	0.0004215*	0.34(0.14, 0.84)
**Hypertension**	48 (55.2)	66 (68)	39 (54.2)	51 (66.2)	0.3762	1.08(0.51, 2.29)
**Diabetes mellitus**	13 (14.9)	19 (19.6)	11 (15.3)	17 (22.1)	0.319	1.33(0.56, 3.16)
**Hypercholesterolaemia**	4 (4.6)	9 (9.3)	13 (18.1)	25 (32.5)	1.902e-08*	7.92(2.43, 25.8)
**Atrial fibrillation**	1 (1.1)	3 (3.1)	1 (1.4)	6 (7.8)	0.02366*	6.7(0.73, 61.31)
**Myocardial infarction**	2 (2.3)	2 (2.1)	2 (2.8)	5 (6.5)	0.1014	2.73(0.49, 15.34)
**TIA**	5 (5.7)	4 (4.1)	0 (0)	1 (1.3)	0.01774*	0.16(0.02, 1.41)
Premorbid medication						
**Antihypertensive**	24 (27.6)	44 (45.4)	26 (36.1)	27 (35.1)	0.5399	1.13(0.56, 2.26)
**Anti-diabetic**	13 (14.9)	19 (19.6)	7 (9.7)	12 (15.6)	0.6182	0.8(0.32, 2.01)
**Cholesterol-lowering**	4 (4.6)	6 (6.2)	15 (20.8)	20 (26)	5.279e-07*	7.13(2.14, 23.76)
**Antiplatelet**	2 (2.3)	7 (7.2)	2 (2.8)	1 (1.3)	0.3586	0.38(0.03, 4.24)
**Anticoagulant**	1 (1.1)	3 (3.1)	2 (2.8)	9 (11.7)	0.0009144*	10.6(1.29, 87.16)

OR indicates odds ratio; CI, confidence interval; and TIA, transient ischaemic attack. † Cochran-Armitage tests for trend. ‡ 2013-2018 with reference to 1995-2000 after adjusting for age and ethnicity

Table S8 Trends in risk factors in overall HS patients <55 years

Young	1995-2000	2001-2006	2007-2012	2013-2018	P-value (trend)†	Adjusted model‡ OR (95% CI)
Premorbid risk factors						
**Current drinker**	70 (67.3)	76 (58)	41 (54.7)	33 (39.8)	0.0001111*	0.28(0.12, 0.61)
**Smoker**	53 (51)	47 (35.9)	29 (38.7)	15 (18.1)	7.844e-06*	0.26(0.12, 0.58)
**Hypertension**	37 (35.6)	55 (42)	27 (36)	32 (38.6)	0.8926	0.73(0.37, 1.45)
**Diabetes mellitus**	8 (7.7)	4 (3.1)	10 (13.3)	10 (12)	0.06838	1.22(0.44, 3.39)
**Hypercholesterolaemia**	4 (3.8)	7 (5.3)	5 (6.7)	10 (12)	0.02286*	2.47(0.71, 8.66)
**Atrial fibrillation**	1 (1)	2 (1.5)	1 (1.3)	0 (0)	0.4789	N/A
**Myocardial infarction**	2 (1.9)	2 (1.5)	1 (1.3)	2 (2.4)	0.8307	1.16(0.16, 8.32)
**TIA**	3 (2.9)	3 (2.3)	1 (1.3)	0 (0)	0.08955	N/A
Premorbid medication						
**Antihypertensive**	19 (18.3)	31 (23.7)	15 (20)	13 (15.7)	0.5158	0.55(0.24, 1.25)
**Anti-diabetic**	8 (7.7)	4 (3.1)	8 (10.7)	4 (4.8)	0.9287	0.37(0.1, 1.33)
**Cholesterol-lowering**	2 (1.9)	4 (3.1)	7 (9.3)	6 (7.2)	0.02776*	2.74(0.58, 13.07)
**Antiplatelet**	2 (1.9)	1 (0.8)	1 (1.3)	1 (1.2)	0.7538	0.35(0.03, 4.28)
**Anticoagulant**	2 (1.9)	0 (0)	0 (0)	4 (4.8)	0.1307	N/A

OR indicates odds ratio; CI, confidence interval; and TIA, transient ischaemic attack. † Cochran-Armitage tests for trend. ‡ 2013-2018 with reference to 1995-2000 after adjusting for age and ethnicity

Table S9 Trends in risk factors in overall HS patients 55+ years

**Old**	**1995-2000**	**2001-2006**	**2007-2012**	**2013-2018**	P-value (trend)†	Adjusted model‡ OR (95% CI)
Premorbid risk factors						
**Current drinker**	146 (59.1)	111 (50)	50 (36.5)	41 (34.2)	7.183e-05*	0.42(0.24, 0.73)
**Smoker**	87 (35.2)	53 (23.9)	19 (13.9)	14 (11.7)	1.008e-05*	0.3(0.15, 0.59)
**Hypertension**	141 (57.1)	140 (63.1)	83 (60.6)	71 (59.2)	0.8638	0.94(0.57, 1.54)
**Diabetes mellitus**	22 (8.9)	39 (17.6)	22 (16.1)	24 (20)	0.05054*	2.24(1.17, 4.28)
**Hypercholesterolaemia**	4 (1.6)	26 (11.7)	47 (34.3)	38 (31.7)	2.558e-10*	10.81(4.02, 29.08)
**Atrial fibrillation**	26 (10.5)	22 (9.9)	22 (16.1)	25 (20.8)	0.01806*	3.14(1.67, 5.91)
**Myocardial infarction**	17 (6.9)	15 (6.8)	11 (8)	8 (6.7)	0.9555	1.07(0.44, 2.63)
**TIA**	21 (8.5)	11 (5)	6 (4.4)	5 (4.2)	0.1835	0.43(0.15, 1.21)
Premorbid medication						
**Antihypertensive**	58 (23.5)	90 (40.5)	39 (28.5)	34 (28.3)	0.9039	1.13(0.66, 1.94)
**Anti-diabetic**	22 (8.9)	40 (18)	11 (8)	19 (15.8)	0.4695	1.61(0.81, 3.21)
**Cholesterol-lowering**	6 (2.4)	24 (10.8)	46 (33.6)	34 (28.3)	8.849e-09*	9.77(4.13, 23.12)
**Antiplatelet**	33 (13.4)	20 (9)	9 (6.6)	1 (0.8)	0.0006487*	0.06(0.01, 0.51)
**Anticoagulant**	12 (4.9)	17 (7.7)	20 (14.6)	24 (20)	0.0002814*	5.98(2.85, 12.54)

OR indicates odds ratio; CI, confidence interval; and TIA, transient ischaemic attack. † Cochran-Armitage tests for trend. ‡ 2013-2018 with reference to 1995-2000 after adjusting for age and ethnicity

Table S10 Trends in risk factors in PICH male patients

Male	**1995-2000** N= 144	**2001-2006** N= 141	**2007-2012** N= 89	**2013-2018** N= 85	P-value (trend)†	Adjusted model‡ OR (95% CI)
Premorbid risk factors						
**Current drinker**	97 (67.4)	81 (57.4)	44 (49.4)	41 (48.2)	0.001*	0.43(0.22, 0.82)
**Smoker**	66 (45.8)	39 (27.7)	26 (29.2)	9 (10.6)	<0.0001*	0.2(0.09, 0.46)
**Hypertension**	72 (50)	82 (58.2)	50 (56.2)	45 (52.9)	0.629	1.03(0.57, 1.87)
**Diabetes mellitus**	17 (11.8)	24 (17)	14 (15.7)	14 (16.5)	0.3549	1.5(0.67, 3.34)
**Hypercholesterolaemia**	3 (2.1)	17 (12.1)	25 (28.1)	19 (22.4)	<0.0001*	8.87(2.56, 30.72)
**Atrial fibrillation**	12 (8.3)	11 (7.8)	7 (7.9)	13 (15.3)	0.1325	3.84(1.52, 9.75)
**Myocardial infarction**	11 (7.6)	6 (4.3)	8 (9)	6 (7.1)	0.8332	1.09(0.36, 3.36)
**TIA**	10 (6.9)	8 (5.7)	4 (4.5)	2 (2.4)	0.1228	0.35(0.07, 1.62)
Premorbid medication						
**Antihypertensive**	32 (22.2)	51 (36.2)	29 (32.6)	21 (24.7)	0.5916	1.12(0.57, 2.19)
**Anti-diabetic**	17 (11.8)	24 (17)	10 (11.2)	9 (10.6)	0.6081	0.83(0.34, 2.04)
**Cholesterol-lowering**	5 (3.5)	14 (9.9)	28 (31.5)	18 (21.2)	<0.0001*	6.81(2.38, 19.48)
**Antiplatelet**	13 (9)	14 (9.9)	6 (6.7)	1 (1.2)	0.03*	0.15(0.02, 1.21)
**Anticoagulant**	7 (4.9)	6 (4.3)	10 (11.2)	14 (16.5)	<0.001*	6.6(2.42, 18.01)

OR indicates odds ratio; CI, confidence interval; and TIA, transient ischaemic attack. † Cochran-Armitage tests for trend. ‡ 2013-2018 with reference to 1995-2000 after adjusting for age and ethnicity

Table S11 Trends in risk factors in PICH female patients

Female	**1995-2000** N= 107	**2001-2006** N= 105	**2007-2012** N= 74	**2013-2018** N= 66	P-value (trend)†	Adjusted model‡ OR (95% CI)
Premorbid risk factors						
**Current drinker**	44 (41.1)	39 (37.1)	21 (28.4)	16 (24.2)	0.01*	0.53(0.25, 1.14)
**Smoker**	24 (22.4)	15 (14.3)	9 (12.2)	7 (10.6)	0.03*	0.48(0.19, 1.21)
**Hypertension**	67 (62.6)	74 (70.5)	41 (55.4)	36 (54.5)	0.1226	0.61(0.3, 1.24)
**Diabetes mellitus**	10 (9.3)	12 (11.4)	15 (20.3)	16 (24.2)	<0.01*	2.65(1.07, 6.53)
**Hypercholesterolaemia**	3 (2.8)	12 (11.4)	16 (21.6)	18 (27.3)	<0.0001*	6.69(1.96, 22.86)
**Atrial fibrillation**	12 (11.2)	9 (8.6)	14 (18.9)	9 (13.6)	0.259	2.01(0.74, 5.45)
**Myocardial infarction**	5 (4.7)	6 (5.7)	3 (4.1)	4 (6.1)	0.8349	1.48(0.37, 5.92)
**TIA**	12 (11.2)	6 (5.7)	3 (4.1)	0 (0)	<0.01*	0(0, 0)
Premorbid medication						
**Antihypertensive**	31 (29)	45 (42.9)	15 (20.3)	15 (22.7)	0.09149	0.62(0.29, 1.32)
**Anti-diabetic**	10 (9.3)	13 (12.4)	6 (8.1)	10 (15.2)	0.4326	1.3(0.48, 3.49)
**Cholesterol-lowering**	3 (2.8)	11 (10.5)	15 (20.3)	13 (19.7)	<0.0001*	5.87(1.62, 21.33)
**Antiplatelet**	20 (18.7)	7 (6.7)	4 (5.4)	1 (1.5)	<0.0001*	0.07(0.01, 0.56)
**Anticoagulant**	4 (3.7)	9 (8.6)	9 (12.2)	12 (18.2)	<0.01*	7.04(2.16, 22.97)

OR indicates odds ratio; CI, confidence interval; and TIA, transient ischaemic attack. † Cochran-Armitage tests for trend. ‡ 2013-2018 with reference to 1995-2000 after adjusting for age and ethnicity

Table S12 Trends in risk factors in PICH white patients

**White**	**1995-2000** N=170	**2001-2006** N=138	**2007-2012** N=87	**2013-2018** N=74	P-value (trend)†	Adjusted model‡ OR (95% CI)
Premorbid risk factors						
**Current drinker**	100 (58.8)	71 (51.4)	36 (41.4)	34 (45.9)	0.02826*	0.5(0.26, 0.96)
**Smoker**	68 (40)	29 (21)	19 (21.8)	6 (8.1)	<0.0001*	0.16(0.06, 0.39)
**Hypertension**	92 (54.1)	81 (58.7)	47 (54)	28 (37.8)	0.0173*	0.54(0.3, 0.98)
**Diabetes mellitus**	12 (7.1)	11 (8)	13 (14.9)	10 (13.5)	0.05938	2.16(0.89, 5.28)
**Hypercholesterolaemia**	2 (1.2)	15 (10.9)	22 (25.3)	14 (18.9)	<0.0001*	6.95(1.9, 25.41)
**Atrial fibrillation**	22 (12.9)	15 (10.9)	19 (21.8)	14 (18.9)	0.0799	2.09(0.96, 4.57)
**Myocardial infarction**	13 (7.6)	7 (5.1)	8 (9.2)	4 (5.4)	0.8158	0.79(0.24, 2.57)
**TIA**	16 (9.4)	7 (5.1)	7 (8)	1 (1.4)	0.051	0.14(0.02, 1.11)
Premorbid medication						
**Antihypertensive**	38 (22.4)	45 (32.6)	17 (19.5)	10 (13.5)	0.7445	0.61(0.29, 1.31)
**Anti-diabetic**	12 (7.1)	12 (8.7)	5 (5.7)	6 (8.1)	0.9806	1.27(0.45, 3.59)
**Cholesterol-lowering**	4 (2.4)	13 (9.4)	25 (28.7)	12 (16.2)	<0.0001*	5.3(1.84, 15.25)
**Antiplatelet**	29 (17.1)	10 (7.2)	7 (8)	1 (1.4)	<0.001*	0.07(0.01, 0.56)
**Anticoagulant**	10 (5.9)	11 (8)	15 (17.2)	13 (17.6)	<0.001*	4.54(1.86, 11.08)

OR indicates odds ratio; CI, confidence interval; and TIA, transient ischaemic attack. † Cochran-Armitage tests for trend. ‡ 2013-2018 with reference to 1995-2000 after adjusting for age and ethnicity

Table S13 Trends in risk factors in PICH black patients

**Black**	**1995-2000** N= 55	**2001-2006** N=72	**2007-2012** N= 56	**2013-2018** N= 56	P-value (trend)†	Adjusted model‡ OR (95% CI)
Premorbid risk factors						
**Current drinker**	31 (56.4)	34 (47.2)	25 (44.6)	17 (30.4)	0.0003*	0.41(0.17, 1)
**Smoker**	12 (21.8)	16 (22.2)	14 (25)	3 (5.4)	0.0074*	0.38(0.11, 1.35)
**Hypertension**	36 (65.5)	54 (75)	32 (57.1)	40 (71.4)	0.997	1.07(0.43, 2.71)
**Diabetes mellitus**	12 (21.8)	18 (25)	9 (16.1)	15 (26.8)	0.7485	1.24(0.49, 3.14)
**Hypercholesterolaemia**	3 (5.5)	8 (11.1)	10 (17.9)	19 (33.9)	<0.0001*	7.52(1.87, 30.2)
**Atrial fibrillation**	1 (1.8)	3 (4.2)	1 (1.8)	5 (8.9)	0.03418*	5.04(0.52, 48.6)
**Myocardial infarction**	2 (3.6)	2 (2.8)	2 (3.6)	5 (8.9)	0.07928	2.45(0.42, 14.19)
**TIA**	5 (9.1)	4 (5.6)	0 (0)	1 (1.8)	0.02*	0.15(0.02, 1.4)
Premorbid medication						
**Antihypertensive**	20 (36.4)	35 (48.6)	21 (37.5)	21 (37.5)	0.7999	1.02(0.46, 2.3)
**Anti-diabetic**	12 (21.8)	18 (25)	5 (8.9)	10 (17.9)	0.2071	0.68(0.25, 1.82)
**Cholesterol-lowering**	4 (7.3)	5 (6.9)	11 (19.6)	16 (28.6)	<0.001*	5.08(1.48, 17.44)
**Antiplatelet**	2 (3.6)	7 (9.7)	2 (3.6)	1 (1.8)	0.3161	0.39(0.03, 4.42)
**Anticoagulant**	1 (1.8)	3 (4.2)	2 (3.6)	8 (14.3)	<0.01*	9.46(1.12, 79.84)

OR indicates odds ratio; CI, confidence interval; and TIA, transient ischaemic attack. † Cochran-Armitage tests for trend. ‡ 2013-2018 with reference to 1995-2000 after adjusting for age and ethnicity

Table S14 Trends in risk factors in PICH patients <55 years

**Young**	**1995-2000** N= 49	**2001-2006** N=70	**2007-2012** N=49	**2013-2018** N=56	P-value (trend)†	Adjusted model‡ OR (95% CI)
Premorbid risk factors						
**Current drinker**	28 (57.1)	32 (45.7)	24 (49)	24 (42.9)	0.077	0.51(0.2, 1.31)
**Smoker**	20 (40.8)	15 (21.4)	18 (36.7)	8 (14.3)	0.498	0.39(0.14, 1.08)
**Hypertension**	25 (51)	37 (52.9)	22 (44.9)	26 (46.4)	0.331	0.65(0.27, 1.54)
**Diabetes mellitus**	7 (14.3)	4 (5.7)	8 (16.3)	10 (17.9)	0.163	1.21(0.4, 3.68)
**Hypercholesterolaemia**	2 (4.1)	5 (7.1)	5 (10.2)	8 (14.3)	0.008*	3.04(0.63, 14.62)
**Atrial fibrillation**	1 (2)	1 (1.4)	1 (2)	0 (0)	NA	N/A
**Myocardial infarction**	1 (2)	0 (0)	1 (2)	2 (3.6)	NA	N/A
**TIA**	3 (6.1)	3 (4.3)	1 (2)	0 (0)	0.0526	1(0, 0)
Premorbid medication						
**Antihypertensive**	14 (28.6)	21 (30)	14 (28.6)	9 (16.1)	0.194	0.41(0.16, 1.1)
**Anti-diabetic**	7 (14.3)	4 (5.7)	6 (12.2)	4 (7.1)	0.4616	0.36(0.1, 1.39)
**Cholesterol-lowering**	2 (4.1)	3 (4.3)	6 (12.2)	5 (8.9)	0.1532	1.97(0.37, 10.4)
**Antiplatelet**	1 (2)	1 (1.4)	1 (2)	1 (1.8)	1	0.67(0.04, 11.86)
**Anticoagulant**	2 (4.1)	0 (0)	0 (0)	4 (7.1)	0.2546	N/A

OR indicates odds ratio; CI, confidence interval; and TIA, transient ischaemic attack. † Cochran-Armitage tests for trend. ‡ 2013-2018 with reference to 1995-2000 after adjusting for age and ethnicity

Table S15 Trends in risk factors in PICH patients 55+ years

**Old**	**1995-2000** N=202	**2001-2006** N=176	**2007-2012** N=114	**2013-2018** N=95	P-value (trend)†	Adjusted model‡ OR (95% CI)
Premorbid risk factors						
**Current drinker**	113 (55.9)	88 (50)	41 (36)	33 (34.7)	<0.001*	0.46(0.25, 0.83)
**Smoker**	70 (34.7)	39 (22.2)	17 (14.9)	8 (8.4)	<0.001*	0.23(0.1, 0.51)
**Hypertension**	114 (56.4)	119 (67.6)	69 (60.5)	55 (57.9)	0.907	0.91(0.52, 1.58)
**Diabetes mellitus**	20 (9.9)	32 (18.2)	21 (18.4)	20 (21.1)	0.045*	2.18(1.08, 4.42)
**Hypercholesterolaemia**	4 (2)	24 (13.6)	36 (31.6)	29 (30.5)	<0.001*	9.36(3.33, 26.3)
**Atrial fibrillation**	23 (11.4)	19 (10.8)	20 (17.5)	22 (23.2)	<0.01*	3.2(1.62, 6.32)
**Myocardial infarction**	15 (7.4)	12 (6.8)	10 (8.8)	8 (8.4)	0.685	1.19(0.48, 2.94)
**TIA**	19 (9.4)	11 (6.3)	6 (5.3)	2 (2.1)	0.01*	0.2(0.05, 0.9)
Premorbid medication						
**Antihypertensive**	49 (24.3)	75 (42.6)	30 (26.3)	27 (28.4)	0.492	1.12(0.62, 2.03)
**Anti-diabetic**	20 (9.9)	33 (18.8)	10 (8.8)	15 (15.8)	0.4659	1.44(0.67, 3.06)
**Cholesterol-lowering**	6 (3)	22 (12.5)	37 (32.5)	26 (27.4)	<0.0001*	7.9(3.21, 19.46)
**Antiplatelet**	32 (15.8)	20 (11.4)	9 (7.9)	1 (1.1)	<0.0001*	0.06(0.01, 0.52)
**Anticoagulant**	9 (4.5)	15 (8.5)	19 (16.7)	22 (23.2)	<0.0001*	7.48(3.28, 17.05)

OR indicates odds ratio; CI, confidence interval; and TIA, transient ischaemic attack. † Cochran-Armitage tests for trend. ‡ 2013-2018 with reference to 1995-2000 after adjusting for age and ethnicity

Table S16 Trends in risk factors in SAH patients

All SAH	**1995-2000**	**2001-2006**	**2007-2012**	**2013-2018**	P-value (trend)†	Adjusted model‡ OR (95% CI)
Premorbid risk factors						
**Current drinker**	75 (75)	67 (62.6)	26 (53.1)	17 (32.7)	7.94e-10*	0.92(0.16, 0.06)
**Smoker**	50 (50)	46 (43)	13 (26.5)	13 (25)	2.069e-05*	0.94(0.33, 0.14)
**Hypertension**	39 (39)	39 (36.4)	19 (38.8)	22 (42.3)	0.5743	1.67(0.89, 0.41)
**Diabetes mellitus**	3 (3)	7 (6.5)	3 (6.1)	4 (7.7)	0.193	7.24(1.88, 0.38)
**Hypercholesterolaemia**	2 (2)	4 (3.7)	11 (22.4)	11 (21.2)	2.233e-07*	44.84(10.66, 2.2)
**Atrial fibrillation**	3 (3)	4 (3.7)	2 (4.1)	3 (5.8)	0.332	N/A
**Myocardial infarction**	3 (3)	5 (4.7)	1 (2)	0 (0)	0.09108	N/A
**TIA**	2 (2)	0 (0)	0 (0)	3 (5.8)	0.06689	N/A
Premorbid medication						
**Antihypertensive**	14 (14)	25 (23.4)	10 (20.4)	11 (21.2)	0.2986	2.51(1.2, 0.47)
**Anti-diabetic**	3 (3)	7 (6.5)	3 (6.1)	4 (7.7)	0.193	7.89(2.35, 0.45)
**Cholesterol-lowering**	0 (0)	3 (2.8)	10 (20.4)	9 (17.3)	2.556e-07*	N/A
**Antiplatelet**	2 (2)	0 (0)	0 (0)	0 (0)	0.05715	N/A
**Anticoagulant**	3 (3)	2 (1.9)	1 (2)	2 (3.8)	0.707	N/A

OR indicates odds ratio; CI, confidence interval; and TIA, transient ischaemic attack. † Cochran-Armitage tests for trend. ‡ 2013-2018 with reference to 1995-2000 after adjusting for age and ethnicity

Table S17 Proportions in risk factors in SAH male patients

Male	1995-2000	2001-2006	2007-2012	2013-2018
Premorbid risk factors				
**Current drinker**	36 (80)	36 (70.6)	11 (61.1)	11 (47.8)
**Smoker**	25 (55.6)	25 (49)	5 (27.8)	8 (34.8)
**Hypertension**	21 (46.7)	16 (31.4)	6 (33.3)	11 (47.8)
**Diabetes mellitus**	2 (4.4)	3 (5.9)	1 (5.6)	2 (8.7)
**Hypercholesterolaemia**	1 (2.2)	3 (5.9)	5 (27.8)	5 (21.7)
**Atrial fibrillation**	2 (4.4)	2 (3.9)	2 (11.1)	2 (8.7)
**Myocardial infarction**	1 (2.2)	4 (7.8)	1 (5.6)	0 (0)
**TIA**	1 (2.2)	0 (0)	0 (0)	3 (13)
Premorbid medication				
**Antihypertensive**	7 (15.6)	11 (21.6)	3 (16.7)	6 (26.1)
**Anti-diabetic**	2 (4.4)	3 (5.9)	1 (5.6)	2 (8.7)
**Cholesterol-lowering**	0 (0)	2 (3.9)	3 (16.7)	4 (17.4)
**Antiplatelet**	1 (2.2)	0 (0)	0 (0)	0 (0)
**Anticoagulant**	3 (6.7)	0 (0)	1 (5.6)	1 (4.3)

TIA, transient ischaemic attack.

Table S18 Proportions in risk factors in SAH female patients

Female	1995-2000	2001-2006	2007-2012	2013-2018
Premorbid risk factors				
**Current drinker**	39 (70.9)	31 (55.4)	15 (48.4)	6 (20.7)
**Smoker**	25 (45.5)	21 (37.5)	8 (25.8)	5 (17.2)
**Hypertension**	18 (32.7)	23 (41.1)	13 (41.9)	11 (37.9)
**Diabetes mellitus**	1 (1.8)	4 (7.1)	2 (6.5)	2 (6.9)
**Hypercholesterolaemia**	1 (1.8)	1 (1.8)	6 (19.4)	6 (20.7)
**Atrial fibrillation**	1 (1.8)	2 (3.6)	0 (0)	1 (3.4)
**Myocardial infarction**	2 (3.6)	1 (1.8)	0 (0)	0 (0)
**TIA**	1 (1.8)	0 (0)	0 (0)	0 (0)
Premorbid medication				
**Antihypertensive**	7 (12.7)	14 (25)	7 (22.6)	5 (17.2)
**Anti-diabetic**	1 (1.8)	4 (7.1)	2 (6.5)	2 (6.9)
**Cholesterol-lowering**	0 (0)	1 (1.8)	7 (22.6)	5 (17.2)
**Antiplatelet**	1 (1.8)	0 (0)	0 (0)	0 (0)
**Anticoagulant**	0 (0)	2 (3.6)	0 (0)	1 (3.4)

TIA, transient ischaemic attack.

Table S19 Proportions in risk factors in SAH white patients

White	1995-2000	2001-2006	2007-2012	2013-2018
Premorbid risk factors				
**Current drinker**	49 (79)	44 (65.7)	13 (52)	11 (45.8)
**Smoker**	31 (50)	33 (49.3)	9 (36)	7 (29.2)
**Hypertension**	25 (40.3)	23 (34.3)	9 (36)	7 (29.2)
**Diabetes mellitus**	1 (1.6)	5 (7.5)	0 (0)	1 (4.2)
**Hypercholesterolaemia**	1 (1.6)	2 (3)	8 (32)	3 (12.5)
**Atrial fibrillation**	3 (4.8)	4 (6)	2 (8)	2 (8.3)
**Myocardial infarction**	3 (4.8)	4 (6)	1 (4)	0 (0)
**TIA**	1 (1.6)	0 (0)	0 (0)	2 (8.3)
Premorbid medication				
**Antihypertensive**	9 (14.5)	14 (20.9)	4 (16)	3 (12.5)
**Anti-diabetic**	1 (1.6)	5 (7.5)	0 (0)	1 (4.2)
**Cholesterol-lowering**	0 (0)	2 (3)	6 (24)	3 (12.5)
**Antiplatelet**	2 (3.2)	0 (0)	0 (0)	0 (0)
**Anticoagulant**	3 (4.8)	2 (3)	1 (4)	1 (4.2)

TIA, transient ischaemic attack.

Table S20 Proportions in risk factors in SAH black patients

Black	**1995-2000**	**2001-2006**	**2007-2012**	**2013-2018**
Premorbid risk factors				
**Current drinker**	23 (71.9)	15 (60)	10 (62.5)	5 (23.8)
**Smoker**	18 (56.3)	7 (28)	4 (25)	6 (28.6)
**Hypertension**	12 (37.5)	12 (48)	7 (43.8)	11 (52.4)
**Diabetes mellitus**	1 (3.1)	1 (4)	2 (12.5)	2 (9.5)
**Hypercholesterolaemia**	1 (3.1)	1 (4)	3 (18.8)	6 (28.6)
**Atrial fibrillation**	0 (0)	0 (0)	0 (0)	1 (4.8)
**Myocardial infarction**	5 (15.6)	0 (0)	0 (0)	2 (9.5)
**TIA**	0 (0)	0 (0)	0 (0)	0 (0)
Premorbid medication				
**Antihypertensive**	4 (12.5)	9 (36)	5 (31.3)	6 (28.6)
**Anti-diabetic**	1 (3.1)	1 (4)	2 (12.5)	2 (9.5)
**Cholesterol-lowering**	0 (0)	1 (4)	4 (25)	4 (19)
**Antiplatelet**	0 (0)	0 (0)	0 (0)	0 (0)
**Anticoagulant**	0 (0)	0 (0)	0 (0)	1 (4.8)

TIA, transient ischaemic attack.

Table S21 Proportions in risk factors in SAH patients <55 years

Young	1995-2000	2001-2006	2007-2012	2013-2018
Premorbid risk factors				
**Current drinker**	42 (76.4)	44 (72.1)	17 (65.4)	9 (33.3)
**Smoker**	33 (60)	32 (52.5)	11 (42.3)	7 (25.9)
**Hypertension**	12 (21.8)	18 (29.5)	5 (19.2)	6 (22.2)
**Diabetes mellitus**	1 (1.8)	0 (0)	2 (7.7)	0 (0)
**Hypercholesterolaemia**	2 (3.6)	2 (3.3)	0 (0)	2 (7.4)
**Atrial fibrillation**	0 (0)	1 (1.6)	0 (0)	0 (0)
**Myocardial infarction**	1 (1.8)	2 (3.3)	0 (0)	0 (0)
**TIA**	0 (0)	0 (0)	0 (0)	0 (0)
Premorbid medication				
**Antihypertensive**	5 (9.1)	10 (16.4)	1 (3.8)	4 (14.8)
**Anti-diabetic**	1 (1.8)	0 (0)	2 (7.7)	0 (0)
**Cholesterol-lowering**	0 (0)	1 (1.6)	1 (3.8)	1 (3.7)
**Antiplatelet**	1 (1.8)	0 (0)	0 (0)	0 (0)
**Anticoagulant**	0 (0)	0 (0)	0 (0)	0 (0)

TIA, transient ischaemic attack.

Table S22 Proportions in risk factors in SAH patients 55+ years

Old	1995-2000	2001-2006	2007-2012	2013-2018
Premorbid risk factors				
**Current drinker**	33 (73.3)	23 (50)	9 (39.1)	8 (32)
**Smoker**	17 (37.8)	14 (30.4)	2 (8.7)	6 (24)
**Hypertension**	27 (60)	21 (45.7)	14 (60.9)	16 (64)
**Diabetes mellitus**	2 (4.4)	7 (15.2)	1 (4.3)	4 (16)
**Hypercholesterolaemia**	0 (0)	2 (4.3)	11 (47.8)	9 (36)
**Atrial fibrillation**	3 (6.7)	3 (6.5)	2 (8.7)	3 (12)
**Myocardial infarction**	2 (4.4)	3 (6.5)	1 (4.3)	0 (0)
**TIA**	2 (4.4)	0 (0)	0 (0)	3 (12)
Premorbid medication				
**Antihypertensive**	9 (20)	15 (32.6)	9 (39.1)	7 (28)
**Anti-diabetic**	2 (4.4)	7 (15.2)	1 (4.3)	4 (16)
**Cholesterol-lowering**	0 (0)	2 (4.3)	9 (39.1)	8 (32)
**Antiplatelet**	1 (2.2)	0 (0)	0 (0)	0 (0)
**Anticoagulant**	3 (6.7)	2 (4.3)	1 (4.3)	2 (8)

TIA, transient ischaemic attack.
